# Replisome passage through the cohesin ring

**DOI:** 10.1016/j.cell.2025.08.028

**Published:** 2025-09-16

**Authors:** Samson Glaser, Maxim I. Molodtsov, John F.X. Diffley, Frank Uhlmann

**Affiliations:** 1 Chromosome Replication Laboratory, The Francis Crick Institute, London NW1 1AT, UK; 2 Chromosome Segregation Laboratory, The Francis Crick Institute, London NW1 1AT, UK; 3 Mechanobiology and Biophysics Laboratory, The Francis Crick Institute, London NW1 1AT, UK; 4 Department of Physics and Astronomy, University College London, London WC1E 6BT, UK

## Abstract

Following eukaryotic genome replication, the ring-shaped cohesin complex embraces the two newly synthesized sister chromatids, enabling their faithful segregation during cell divisions. Replisome passage through cohesin rings has been envisioned as a fail-safe mechanism that ensures co-entrapment of replication products—whether replisomes can indeed pass through cohesin rings remains unknown. Here, we use biochemical reconstitution and single-molecule fluorescence microscopy to directly visualize replisome-cohesin en-counters. We find that the translocating eukaryotic replicative Cdc45-Mcm2-7-GINS (CMG) helicase, unlike other obstacles of similar size, readily passes through cohesin rings. Fully reconstituted replisomes also pass cohesin rings to leave both replication products trapped inside. Replisome passage is primarily aided by DNA polymerases ***α*** and **ε**, a finding that necessitates re-evaluation of canonical cohesion establishment factor roles. Our findings demonstrate the existence of a simple mechanism that links genome replication with chromosome segregation: replisome passage through cohesin rings.

## Graphical abstract

**Graphical abstract:**
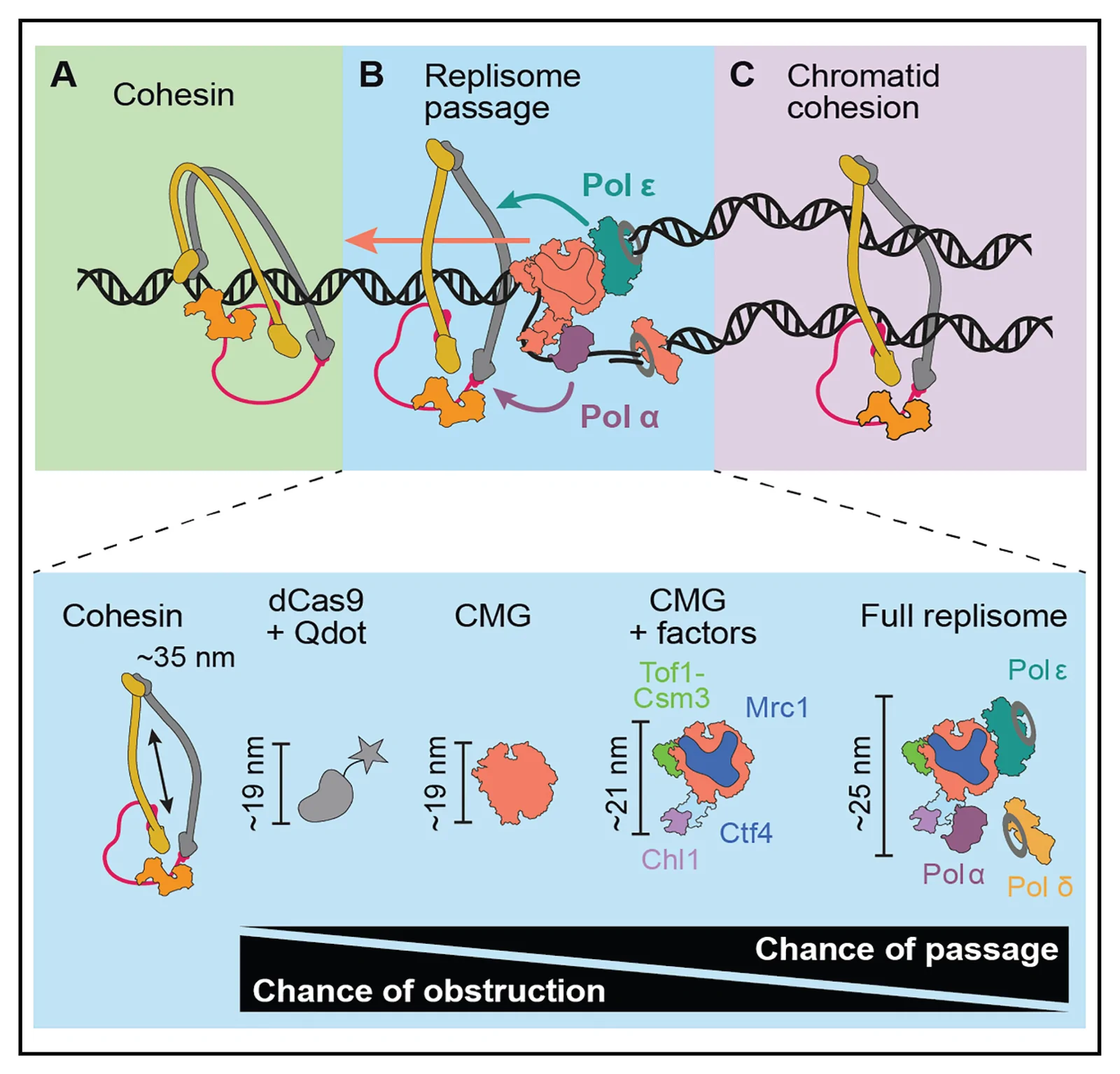

## Introduction

Sister chromatid cohesion provides the counterforce to mitotic spindle forces in the tug of war that aligns chromosomes on the cell equator in preparation for their segregation toward daughter cells when cohesin cleavage triggers anaphase.^1^ The ring-shaped cohesin complex is topologically loaded onto DNA during the G1 phase of the cell division cycle with the help of its Scc2–Scc4 cohesin loader complex.^2–4^ Following DNA replication, the same cohesin rings now embrace the two sister DNAs.^5–8^ As soon as the ring shape of the cohesin complex became apparent,^9,10^ so did an appealing solution for how cohesin transitions from entrapping one DNA to embracing both replication products—replisome passage through the cohesin ring. The cohesin ring diameter is ~35 nm,^9,10^ large enough in principle to allow passage of eukaryotic replisomes, which have a diameter of ~25 nm.^11^ On the other hand, while cohesin readily slides along DNA following topological loading, DNA-bound obstacles as small as ~10 nm are known to form barriers that cohesin struggles to overcome.^12–14^ This small exclusion limit was taken to suggest that the cohesin complex adopts a “collapsed,” or “folded,” conformational state when bound to DNA.^15,16^ Whether, therefore, the replisome can pass through cohesin rings remains uncertain.

In addition to replisome passage through cohesin rings, other possible scenarios have been proposed for how cohesin co-entraps two replication products,^17^ and evidence exists for each of 5618 Cell *188*, 5618–5631, October 2, 2025 © 2025 The Author(s). Published by Elsevier Inc. them. Cohesin might temporarily detach from DNA while the replisome passes and then reload, sequentially, onto both nascent sister DNA products.^7^ Alternatively, cohesin could be newly recruited to DNA replication forks,^3,18^ or replisomes might sometimes push cohesin along DNA before cohesion establishment.^14,19^ Therefore, if we here find that replisomes pass through cohesin rings, this outcome need not be mutually exclusive with complementary mechanisms by which cohesin establishes sister chromatid cohesion. As it stands, our knowledge of the molecular events that take place when a replisome encounters cohesin rings remains limited. Therefore, we here set out to directly visualize biochemically reconstituted replisomes as they meet cohesin rings on DNA by using single-molecule fluorescence microscopy.

In addition to the cohesin rings themselves, a series of replisome components contribute to ensuring that cohesin links sister chromatids following DNA replication. These proteins are known as “cohesion establishment factors.” They include the Tof1-Csm3-Mrc1 (TCM) replisome progression complex,^20–22^ the replisome interaction hub Ctf4 with one of its client proteins, the Chl1 helicase,^23–25^ as well as the PCNA sliding clamp loader Ctf18-RFC.^23,26,27^ Complex genetic relationships between these cohesion establishment factors^28,29^ suggest that more than one reaction contributes to successful cohesion establishment. Tof1, Mrc1, and Chl1 have all been seen engaging in direct protein interactions with the cohesin complex,^30,31^ but how these interactions promote sister chromatid cohesion establishment remains to be understood. Most of the known cohesion establishment factors were identified in genetic screens for non-essential proteins with roles in chromosome stability. Screens for essential components of the cohesion machinery^32–34^ would be unlikely to have identified replisome components, as DNA replication defects would have obscured defective sister chromatid cohesion. How far essential replisome components contribute to cohesion establishment therefore remains incompletely explored.

In addition to sister chromatid co-entrapment, a second essential aspect of cohesion establishment is replication-coupled cohesin acetylation on two conserved lysines of its Smc3 subunit. Cohesin acetylation is required to stabilize sister chromatid linkages that have formed.^35–37^ Again, all the above-mentioned cohesion establishment factors contribute, directly or indirectly, to the cohesin acetylation reaction.^29^

## Results

### CMG as a surmountable cohesin barrier

**Figure 1:**
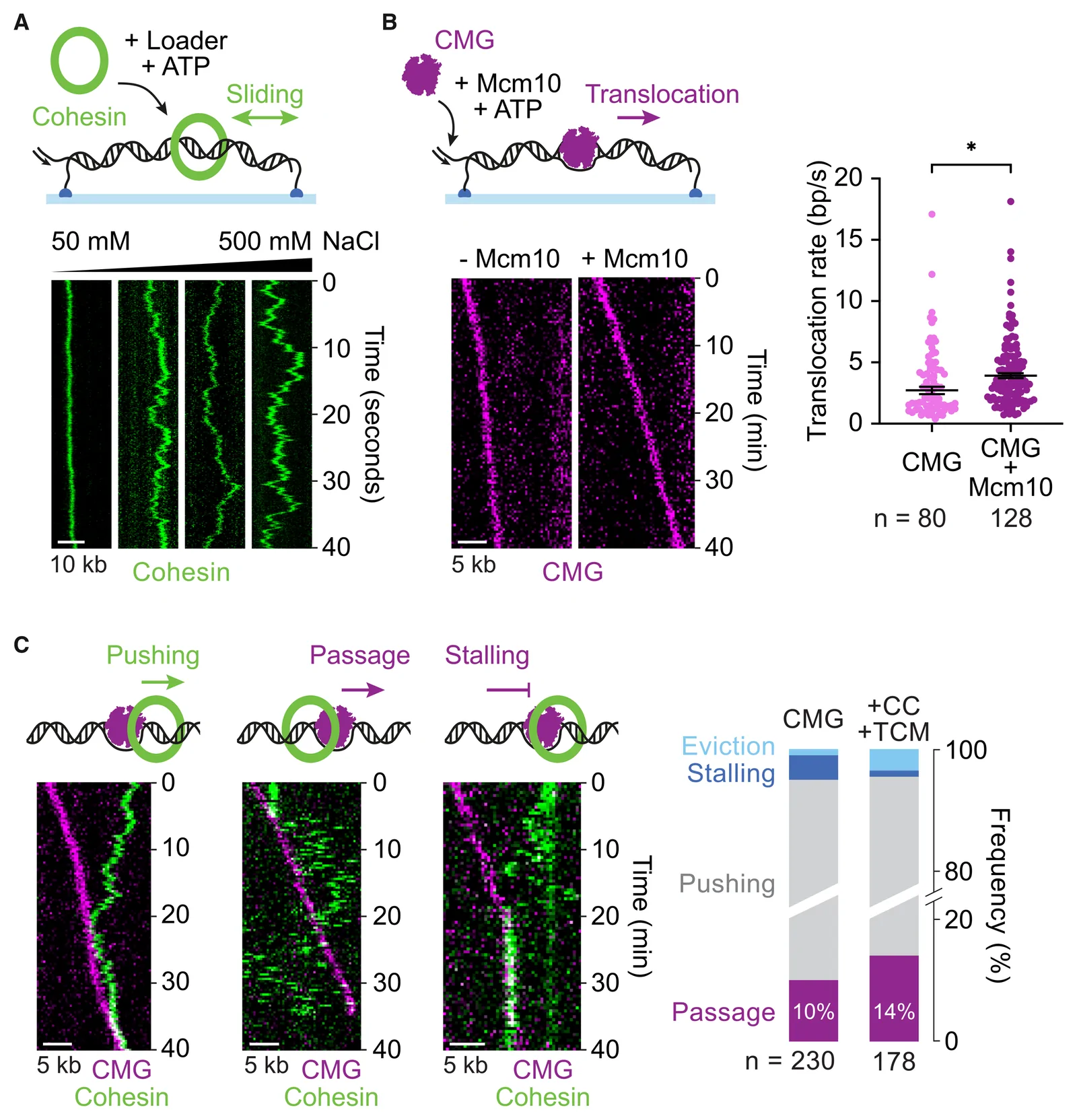
The CMG helicase is a surmountable cohesin barrier (A) Schematic of cohesin loading and sliding, as well as representative kymographs of cohesin loaded onto tethered DNA in a flow cell and imaged at the indicated salt concentrations. See also Figure S1 for ensemble analyses and additional single-molecule experiments with budding yeast cohesin. (B) Schematic of CMG loading and translocation. CMG is loaded at a low ATP concentration, and translocation is initiated by increasing ATP, with or without Mcm10 addition. Representative kymographs of CMG translocation in the presence or absence of Mcm10, as well as distributions of segment translocation rates for CMG without and with Mcm10, are shown. *n* is the number of measured segments, and black lines and error bars represent the weighted mean and standard error (* *p* < 0.001, unpaired t test). See also Figure S2 for ensemble and additional single-molecule analyses of budding yeast CMG. (C) Schematics and representative kymographs from observed outcomes of CMG-cohesin encounters. Frequencies were aggregated from two biological replicates each, using CMG or CMG and added Ctf4-Chl1 (CC) and Tof1-Csm3-Mrc1 (TCM). *n* is the total number of observed encounters (*p*(CMG vs. CMG + CC + TCM) = 0.0527, unpaired t test).

We began by visualizing encounters of the replicative Cdc45-Mcm2-7-GINS (CMG) helicase—the core of the eukaryotic replisome—with cohesin. We purified fluorescently labeled budding yeast cohesin (tetramer complexes consisting of Smc1, Smc3, Scc1, and Scc3) and its Scc2–Scc4 loader complex (Figure S1A). Fluorophore LD655-labeled cohesin exhibited DNA and Scc2–Scc4-stimulated ATPase activity and could be loaded onto DNA in a salt-resistant manner, like unlabeled cohesin (Figures S1B and S1C).^2,38^ Using a microfluidic flow cell, we loaded cohesin onto surface-tethered, stretched linear DNA. Following a high-salt wash to remove non-topologically bound molecules, we visualized cohesin using total internal reflection fluorescence (TIRF) microscopy (Figure 1A). Cohesin was seen as diffraction-limited spots moving along DNA in a diffusive manner. Photobleaching revealed that over 90% of these cohesin spots exhibited single-step photobleaching, indicating that most foci correspond to single cohesin rings (Figure S1D). The diffusion coefficient increased with ionic strength in the imaging buffer. At 500 mM NaCl, it reached *D* = 2.65 ± 0.21 μm^2^/s (mean ± SEM, *n* = 11; Figure S1E), comparable with diffusion coefficients reported for human and fission yeast cohesin^12–14^ or PCNA.^39^ The value is substantially greater than that observed for DNA-binding proteins that associate with DNA by electrostatic interactions,^40^ consistent with loose topological entrapment of the DNA by these protein rings. Topological entrapment was additionally confirmed by DNA cleavage with a restriction enzyme, resulting in cohesin sliding off the free DNA end (Figure S1F).

Next, we purified and fluorescently labeled budding yeast CMG and observed equally efficient bulk DNA unwinding by both unlabeled and labeled CMG (Figures S2A and S2B). To visualize CMG translocation at the single-molecule level, we tethered a linear forked DNA with a free 3′ overhang to the flow cell surface, followed by helicase binding in the presence of a low ATP concentration, and then helicase activation by increasing ATP^41^ (the 3′ overhang contained an annealed primer, inconsequential for CMG translocation experiments but required for later DNA replication assays). This protocol resulted in fluorescently labeled CMG moving unidirectionally along the DNA (Figure 1B). As previously seen,^42^ the addition of the CMG cofactor Mcm10 resulted in an increased translocation rate (Figure S2C). In this experimental setup, unwound DNA reanneals behind the translocating CMG. To confirm that CMG un-winds DNA, we included the single-strand binding protein RPA and the DNA stain SYTOX Orange, which preferentially binds double-stranded DNA. Over 80% of translocation events were now accompanied by loss of the SYTOX Orange signal, confirming that the CMG helicase unwinds DNA during translocation (Figure S2D). In the following, we performed experiments without RPA, resulting in CMG movement in the 3′–5′ direction along the forked strand, which then reannealed.

To visualize cohesin-CMG encounters, we first loaded cohesin onto the forked DNA, then bound and activated the CMG helicase. Note that cohesin mobility along DNA is typically faster than CMG translocation (compare the time scales between Figures 1A and 1B), making cohesin trajectories sometimes appear disjointed on CMG kymographs. When a translocating CMG encountered cohesin, we observed one of four outcomes (Figure 1C). In most cases, (1) CMG appeared to push cohesin or to confine its available space for diffusion (“pushing”). However, in a fraction of instances, (2) cohesin was passed by CMG upon encounter, and cohesin continued diffusive motion along DNA on the other side (“passage,” 23 out of 230 events). Other lower frequency events were (3) apparent cohesin eviction (“eviction”) or (4) CMG stalling upon encounter (“stalling”). While the passage frequency of 10% was low, we note that passage was never previously observed across an approximately similarly sized quantum dot (CMG: ~18.9 nm, quantum dot: ~19.5 nm). ^12,43^

To explore the physiological relevance of cohesin passage events, we investigated whether cohesion establishment factors that associate with CMG (Figure S2A) alter the outcome of CMG-cohesin encounters. Addition of Ctf4-Chl1 (denoted CC) and TCM increased the frequency of passage events (Figure 1C), though this increase did not reach statistical significance (*p* (CMG vs. CMG + CC + TCM) = 0.0527, unpaired t test). By binding CMG, the cohesion establishment factors increase the overall size of the translocating apparatus^25,43,44^ (CMG + CC + TCM: ~21.2 nm). However, rather than augmenting CMG barrier function, if anything, these cohesion establishment factors facilitated cohesin passage. Chl1 and TCM make direct physical contact with cohesin,^30,31^ and these contacts might engage cohesin in ways that facilitate passage.

### Converging CMG helicases pass cohesin

Cohesin is free to slide along bare DNA following topological *in vitro* loading. Cohesin sliding is much more restricted on chromatin substrates by the presence of nucleosomes.^12–14^ In our experiments, we observed that cohesin’s diffusive motion was sometimes constrained, presumably due to unspecific flow cell surface interactions that are a common feature in such experiments. To investigate whether cohesin mobility affects the outcome of CMG-cohesin encounters, we stratified cohesin’s pre-encounter state as either diffusive or static (Figure S2E). This comparison revealed that CMG passage was twice as likely when encountering static as compared with freely diffusing cohesin. This observation suggests that cohesin’s ability to escape from an advancing CMG limits the chance of passage.

**Figure 2:**
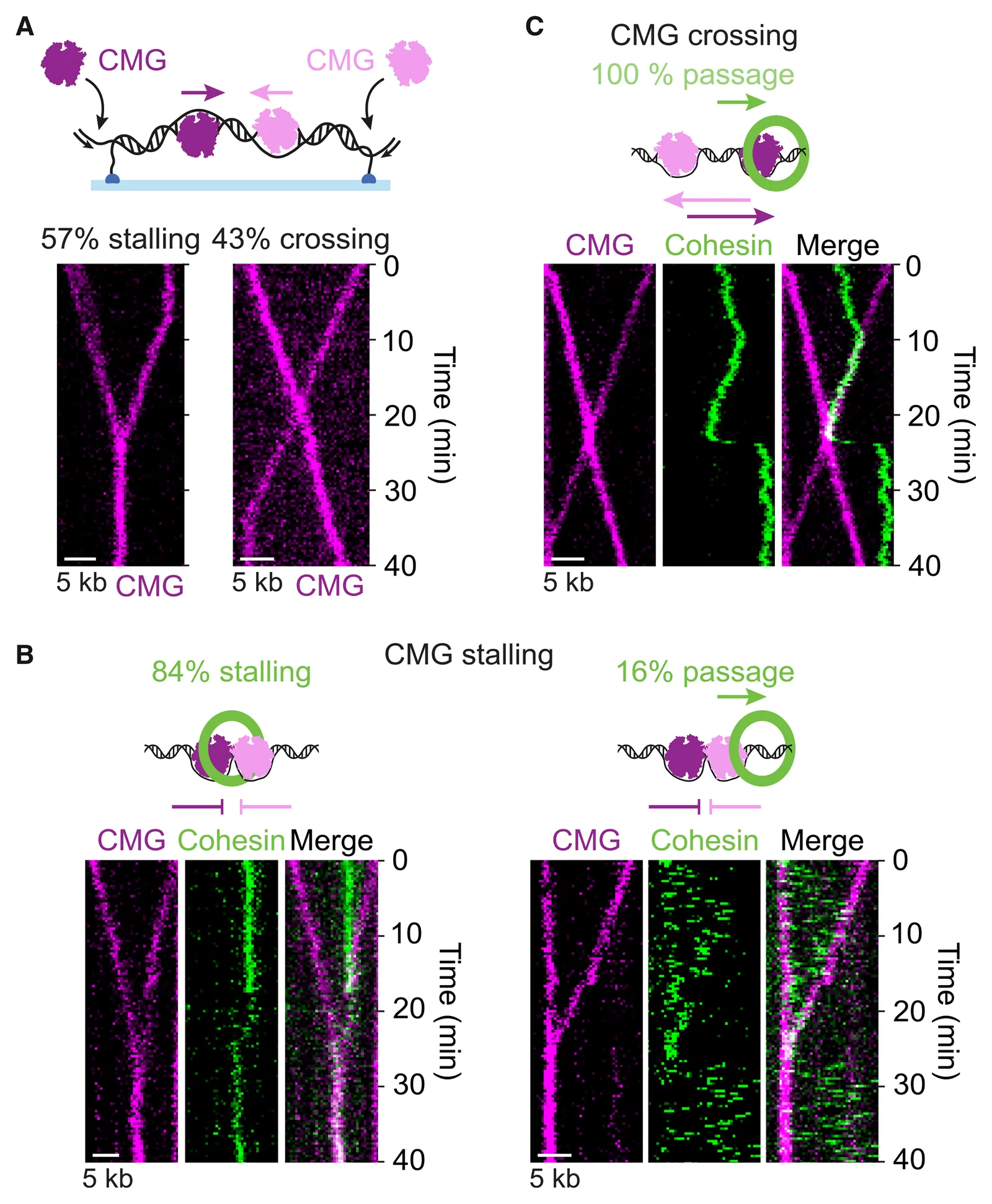
Cohesin passage by converging CMG helicases (A) Schematic and representative kymographs of converging CMG helicases, which either stall (left) or cross each other (right) upon encounter (*n* = 87). (B) Schematics and representative kymographs of cohesin between two stalling CMGs. A cohesin stalling (left) and a cohesin passage (right) event are shown (*n* = 25). (C) Schematic and representative kymograph of cohesin between two crossing CMGs. Cohesin passes one of the CMGs in all cases (*n* = 12).

To investigate the effect of cohesin mobility in a more controlled fashion, we took two approaches. In the first approach, we designed a DNA template featuring 3′ forked overhangs at both ends, allowing the loading of two CMG helicases that approach each other in the 3′–5′ direction on opposite strands. Upon encounter, the two CMGs either stalled 5620 Cell *188*, 5618–5631, October 2, 2025 (57% of events) or passed each other (43% of events) (Figure 2A).

Next, we investigated the fate of cohesin between two converging CMGs. As CMGs approached one another, the range of cohesin movements narrowed up to the point where CMGs met. In cases when CMG movement stalled, cohesin became trapped, but in 16% of cases, cohesin passed one of the two CMGs (Figure 2B). This passage rate was greater than observed with freely mobile cohesin, suggesting that the increased contact time of cohesin and stalled CMGs facilitated passage.

In all those cases where two converging CMG helicases crossed one another, cohesin was pushed by one CMG across the other (Figure 2C). After passing a CMG, cohesin resumed diffusive motion, suggesting that it retained topological DNA embrace during passage. We did not observe cohesin eviction during CMG-CMG passage events, consistent with the idea that cohesin remained topologically intact throughout passage (an idea that we will further explore below). From these observations, we conclude that CMG readily passes cohesin rings if these are prevented from sliding away. A collapsed conformational state of DNA-bound cohesin, which precludes passage of many DNA-bound obstacles,^12–14^ must have been unfolded by the advancing CMG helicase, possibly with the help of cohesion establishment factors that make physical contact with cohesin.

### CMG passage of immobilized cohesin

We used a second experimental approach to study CMG encounters with immobilized cohesins. Following loading onto DNA, we tethered cohesin to the flow cell surface by means of a V5 epitope-specific antibody that we functionalized with a 20 kDa (~150 nm) PEG-biotin linker, recognizing a V5 tag fused to Smc3 (Figure S3A). Introduction of this antibody into the flow cell immobilized previously diffusive cohesin. An unspecific functionalized antibody, or a V5 antibody lacking PEG-biotin (Figure S3B), did not overtly affect cohesin mobility.

**Figure 3:**
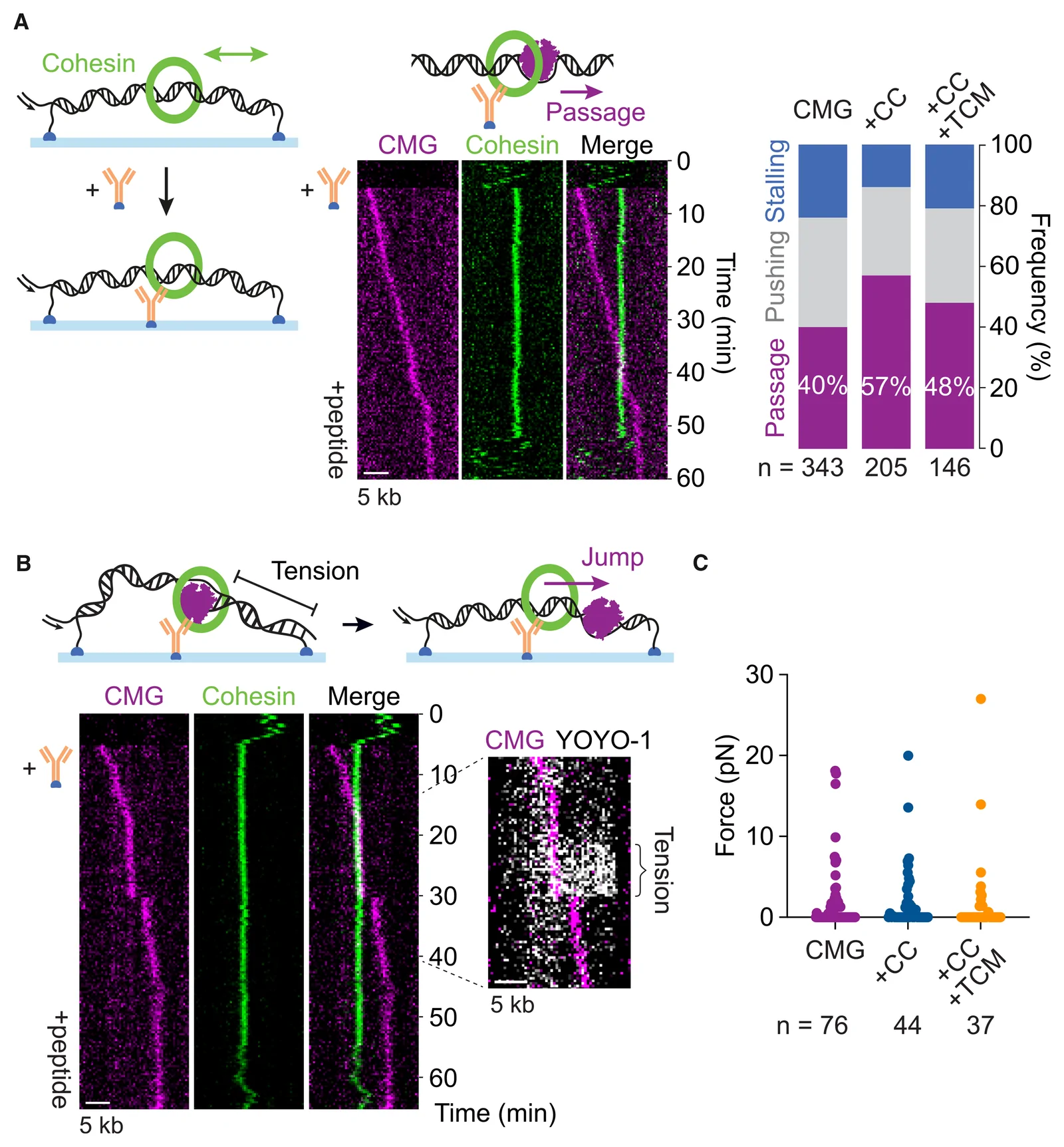
CMG passes immobilized cohesin (A) Schematic of cohesin immobilization using a functionalized antibody and representative kymograph of CMG passing immobilized cohesin. A diffusive cohesin was imaged for 5 min. After cohesin immobilization, CMG was added and activated. Cohesin and CMG were imaged for 40 min before cohesin was mobilized again by peptide elution. Outcomes from three biological replicate experiments were aggregated, as well as two experiments with added Ctf4-Chl1 (+CC) and four experiments with additional Tof1-Csm3 and Mrc1 (+TCM). *n* is the total number of observed encounters (*p*(CMG vs. CMG + CC) = 0.093, *p*(CMG vs. CMG + TCM) = 0.460, unpaired t tests). (B) Schematic and representative kymograph of CMG stalling and tension buildup on encountering immobilized cohesin. Continued CMG translocation during stalling results in DNA stretching, evident by increased YOYO-1 staining ahead of CMG. Upon passage, the stored DNA tension is released, causing the CMG to “jump” forward. (C) Forces generated by CMG leading up to a jump, calculated from the DNA stretch factor and jump size. The force is assumed to be negligible if no jump was observed. *n* is the total number of passage events. See also Figure S3 for additional characterization of CMG-cohesin passage.

We then performed CMG-cohesin encounter experiments with immobilized cohesin. We started the experiment by imaging diffusive cohesin to confirm its topologically loaded state and then introduced the V5 antibody. Next, we added and activated CMG. Cohesin passage now became the most frequent outcome, with 40% of CMG-cohesin encounters resulting in passage (Figure 3B). Other outcomes were cohesin pushing, presumably following cohesin detachment from the antibody, and CMG stalling (Figure S3C). Inclusion of CC, or CC and TCM, cohesion establishment factors again resulted in further small increases in passage frequency, which, however, remained insignificant. Following passage, we introduced V5 peptide into the flow cell, which resulted in cohesin elution from the antibody tether. Diffusive motion resumed along the DNA, indicating that cohesin retained topological DNA association during CMG passage (Figures 3A and S3D). These observations confirm that CMG passage is greatly augmented if cohesin’s ability to escape from the advancing helicase is restrained.

### Force buildup during CMG-cohesin encounters

When we examined CMG traces more closely while passing immobilized cohesin, we noticed transient stalling, followed by small jumps, in approximately half of the encounters (Figures 3B and S3E). No interruptions to CMG progression were Cell *188*, 5618–5631, October 2, 2025 5621 apparent in the other half. CMG stalls and jumps might result if cohesin forms a temporary obstacle, holding up CMG while the helicase continues to reel in DNA. Continuing translocation 5622 Cell *188*, 5618–5631, October 2, 2025 along DNA would lead to tension buildup, which upon passage is released, resulting in rapid DNA relaxation and a visible CMG jump forward. Following the jump, CMG continued to translocate along DNA at similar rates as compared with before passage (Figure S3F).

To explore the possibility that DNA tension accumulates during stalling, we conducted three-color TIRF experiments in which we simultaneously visualized CMG, cohesin, and the DNA stain YOYO-1. DNA tension facilitates dye intercalation, so that increased YOYO-1 staining can serve as an indicator for strain.^45^ The YOYO-1 intensity ahead of CMG, but not behind, increased during stalling and dropped again coincident with the jump (Figure 3C). These observations are suggestive of tension buildup during stalling and release upon cohesin passage. Following jump events, we again used V5 peptide elution to release cohesin from its surface tether. Cohesin resumed diffusive motion along the DNA, confirming that it retained topological DNA embrace also during force-coupled passage.

We observed a positive correlation where longer CMG stalling times led to proportionally larger jumps upon DNA relaxation, consistent with continuous DNA translocation during the stall (Figure S3G). During only short stalls, no increased YOYO-1 signals nor jumps were apparent (Figure S3H), suggesting minimal tension buildup.

Next, we wanted to know how much tension force is generated by CMG before the jumps. From the measured change in DNA segment length in front of the CMG helicase, before and after the jump, and the known DNA force-extension relationship,^46^ we derived the stretching force that preceded individual CMG jump events (Figure 3C; see the STAR Methods for details). This analysis revealed that the vast majority of passage events occurred at well below 20 pN and therefore well below the force required to rupture the cohesin ring at its weakest interface.^8^ These observations are consistent with a mechanical model in which CMG passes through the cohesin ring, but in which passage is sometimes obstructed by cohesin’s folded conformational state.^12^ A passage-competent cohesin shape might be reached in a conformational search, aided by cohesion establishment factors that interact with cohesin, as well as by force buildup by the advancing CMG helicase.

### CMG passage through the cohesin ring

**Figure 4:**
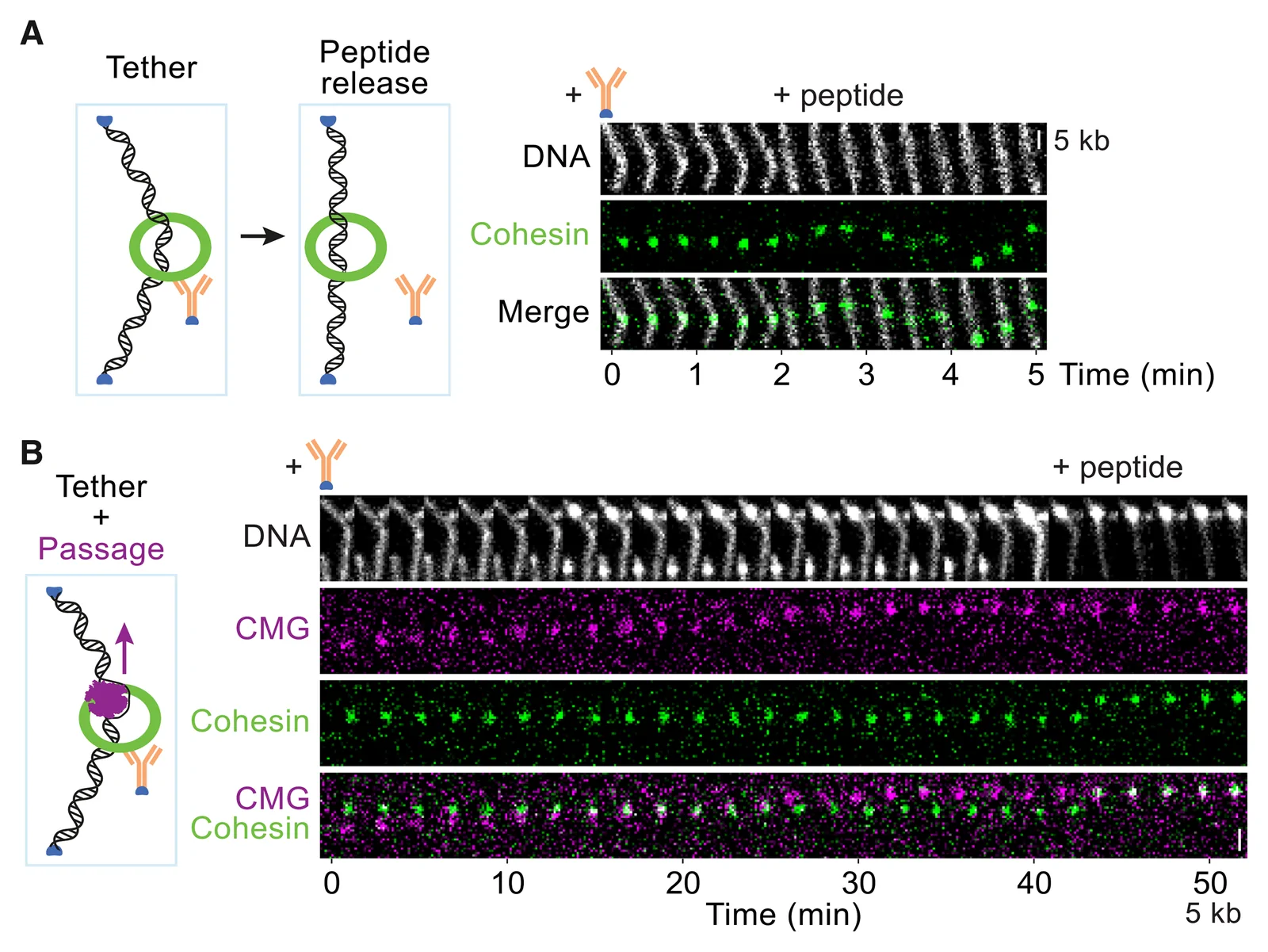
Cohesin retains DNA association during CMG passage (A) Schematic and example time series of side-flow-stretched DNA, tethered by cohesin (Tether). Upon peptide addition, DNA and cohesin are released from the antibody (peptide release). See also Figure S4 for characterization of spontaneous stretched DNA release. (B) Schematic and representative time series of CMG passage along stretched DNA, past immobilized cohesin. Cohesin remains DNA-bound during passage, as well as after peptide elution when DNA regains a straight shape.

The above experiments have established that cohesin retains topologically DNA binding during CMG passage. However, what happens to cohesin at the moment of passage remained unresolved by these observations. Does CMG indeed pass through the intact cohesin ring, or does the ring temporarily open by disengagement of one of its interfaces? To differentiate between these scenarios, we challenged the cohesin-DNA interaction during CMG passage in side-flow experiments. In these experiments, cohesin was loaded onto DNA, and we then applied a strong side flow before tethering cohesin to the flow cell surface using the V5 anti-body (Figure 4A). As a result, after we stopped the side flow, DNA was held by cohesin in an extended V-shape. Cohesin elution from the antibody tether by V5 peptide released the DNA, which regained a linear geometry while cohesin resumed diffusive motion. These observations demonstrate that a topologically loaded cohesin can resist a perpendicular pull on the DNA.

We noticed that around 10% of observed V-shaped DNA tethers spontaneously resolved during the observation period, i.e., DNA suddenly straightened while cohesin itself remained surface-bound. A similar fraction of cohesin detached itself from DNA when similarly incubated without tension (Figure S4). From this observation, we conclude that cohesin sometimes spontaneously unloads from DNA^47^ and that, once released, electrostatic interactions between DNA and cohesin are insufficient to retain the V-shaped DNA geometry.

We next observed CMG translocation along the V-shaped DNA. If cohesin transiently opened during CMG passage, the applied perpendicular pull should result in DNA release and return to its linear conformation. In contrast to this expectation, DNA retained its extended V-shaped geometry during all observed CMG passage events, indicating that DNA remained topologically entrapped by the cohesin ring while CMG traversed (*n* = 4; Figure 4B). Peptide elution of cohesin from the antibody tether after CMG passage eventually allowed DNA recoil. TheseCell *188*, 5618–5631, October 2, 2025 5623 observations suggest that CMG passed through an intact cohesin ring. The alternative explanation, that the cohesin ring opened but DNA remained cohesin-bound by electrostatic interactions, is made unlikely by the above-observed spontaneous detachment events, during which electrostatic cohesin interactions were insufficient to retain DNA. We also know of no direct CMG-cohesin interaction,^30^ which could have maintained contact with an open cohesin ring during passage.

### Establishment of sister chromatid cohesion

**Figure 5:**
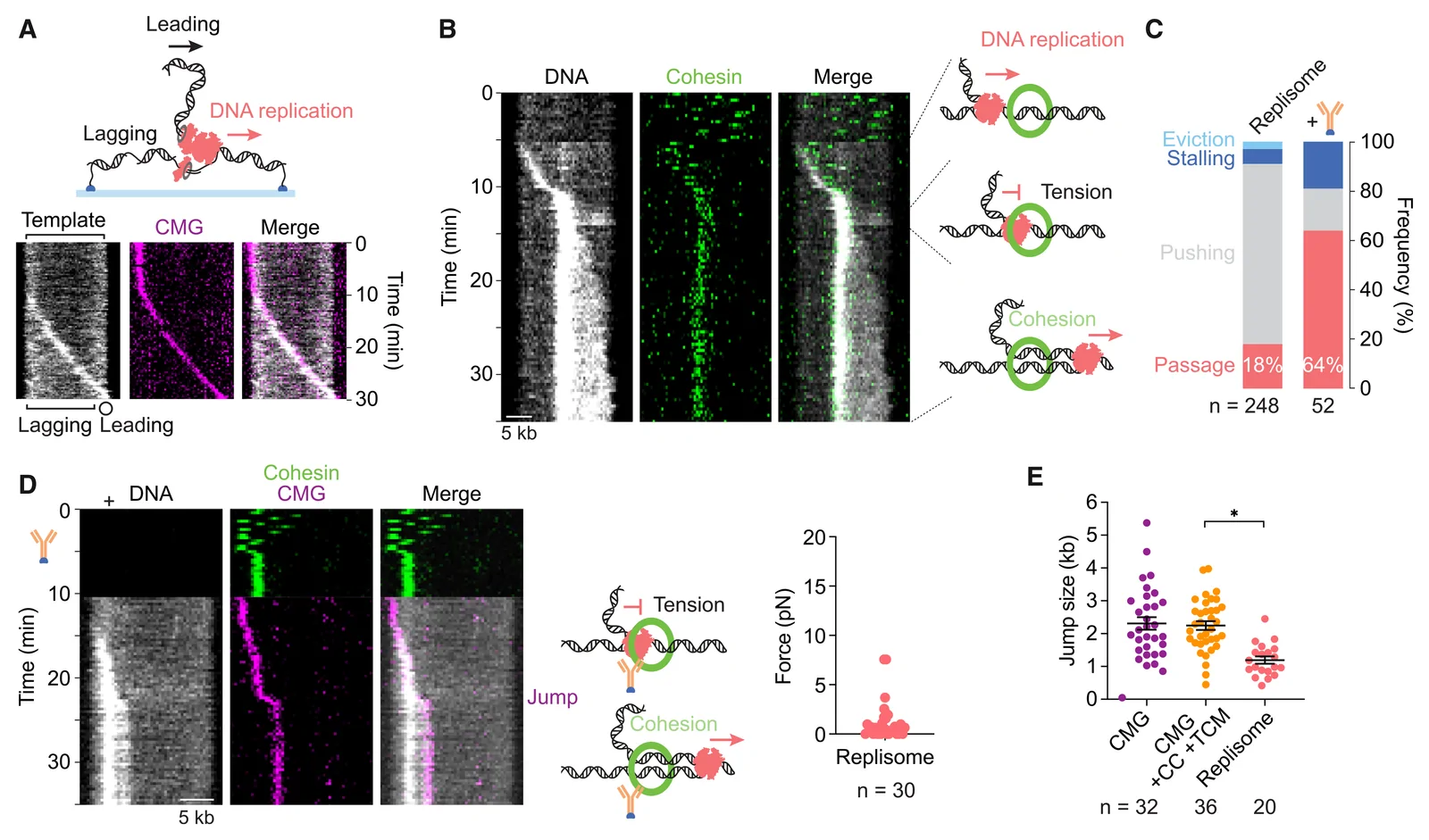
Replisome passage through the cohesin ring (A) Schematic and example kymograph of the DNA replication assay using fluorophore-labeled CMG. The DNA template, the lagging-strand replication product, and the globular-coiled leading-strand product are indicated. Intensity fluctuations of the latter are likely due to diffusive motion in and out of the TIRF excitation volume. See also Figure S5 for the purified replication proteins, as well as replication rates under various conditions. (B) Schematics and representative kymograph of sister chromatid cohesion establishment by replisome passage through the cohesin ring. First, the replisome approaches cohesin. On encounter, a stretch force transiently builds up (uniform SYTOX Orange intensity ahead of the replisome). Following passage, a progressive SYTOX Orange intensity increase results from the two newly synthesized leading and lagging strands. (C) The outcomes of replisome-cohesin encounters were aggregated from 4 biological replicates (replication) and 2 replicates (antibody-immobilized cohesin), and *n* is the total number of observed encounters. See also Figure S6 for additional characterization of single-molecule replisome-cohesin encounters. (D) Schematic and example kymograph of a replisome including LD655-labeled CMG encountering immobilized cohesin. Cohesin was only imaged until 10 min, then excitation was changed to image CMG and DNA. Forces generated in the run-up to jumps were calculated from the DNA stretch factor and jump size. The force is assumed to be negligible if no jump was observed. *n* is the total number of passage events. (E) Comparison of jump sizes during CMG, CMG plus cohesion establishment factors (+CC + TCM), and replisome-cohesin encounters. Black lines and error bars represent means and standard errors (* *p* < 0.0001, unpaired t test). See also Figure S7 for sister chromatid cohesion establishment frequencies at successive positions along the template DNA.

Finally, we visualized fully reconstituted replisomes in the act of replicating our forked DNA substrate^41^ and encountering cohesin. We bound CMG to the primed fork in the presence of the non-hydrolysable ATP analog adenylyl-imidodiphosphate (AMP-PNP) to prevent any onset of DNA unwinding. We then 5624 Cell *188*, 5618–5631, October 2, 2025 added the remaining replisome components Mcm10, Ctf4, Tof1-Csm3, Mrc1, Pol α, Pol δ, Pol ε, RFC, PCNA, and RPA (Figure S5A), exchanged AMP-PNP for ATP, and added the remaining NTPs and dNTPs. DNA replication was visualized using SYTOX Orange, which stains the leading-strand DNA product as a bright spot of increasing intensity that moves along the template (Figure 5A). The lagging-strand product remains tethered to the surface such that the DNA stain behind the replisome remains unchanged from that of the template DNA. Fluorescently labeled CMG co-migrated with the leading-strand DNA intensity, marking the position of DNA unwinding. By tracking the centers of leading-strand foci, we quantified the DNA synthesis rate, revealing a range of rates with an average of 19.9 ± 0.6 bp/s (mean ± standard error; Figure S5B), consistent with previous observations.^41,48^ Omitting TCM from the experiment resulted in substantially lower replication rates, as expected,^41,48^ confirming that these cohesion establishment factors are proficient in fulfilling their replication roles in our assay.

To visualize replisome-cohesin encounters, we loaded cohesin onto the template DNA before replisome assembly and replication initiation. Upon replisome encounters with cohesin, we observed two main outcomes. (1) Cohesin remained ahead of the replisome and most often co-migrated with the advancing leading-strand DNA signal. This outcome can be explained if cohesin either was pushed ahead by the replication machinery or if cohesin was transferred onto the leading-strand DNA product upon encounter (Figure S6A). The spatial resolution of TIRF microscopy, recording both cohesin and the leading-strand DNA product as diffraction-limited spots, is insufficient to distinguish between the two possibilities. Notably, (2) 18% of encounters resulted in cohesin passage by the replisome (Figures 5B and 5C). Replisome passage in turn occurred in two ways, with establishment of sister chromatid cohesion or without (discussed further below). Despite the large size of a complete replisome, the cohesin passage frequency was substantially greater when compared with CMG-only cohesin encounters, suggesting that added replisome components aid cohesin passage. Replisome stalling or cohesin eviction upon encounter remained rare events (Figures 5C and S6B).

Most observed instances when the replisome passed cohesin resulted in the establishment of sister chromatid cohesion (31 out of 42 passage events) (Figures 5B and S6B). Cohesion establishment was manifest by the following observations. The leading-strand DNA that was already synthesized at the time of the encounter remained tethered by cohesin, seen as a mass of constant SYTOX Orange staining intensity that colocalized with cohesin for the remainder of the experiment. The DNA intensity ahead of cohesin, in turn, progressively increased as the replisome continued DNA synthesis, indicative of two juxtaposed leading and lagging strands. These results constitute the live observation of sister chromatid cohesion establishment while a replisome passes a cohesin ring.

Some cohesin encounters did not result in cohesion establishment, or in only transient cohesion establishment, after which the leading-strand product continued travelling along the template DNA, leaving cohesin behind. A possible explanation for passage events without, or only transient, cohesion establishment is the absence of a mechanism that prevents the bare leading-strand DNA product from sliding out of the cohesin ring (Figure S6B). If an initially captured leading-strand product can slide out of the cohesin ring, such escape events should become rarer the more leading-strand DNA is synthesized before the cohesin encounter. Indeed, stratification of replisome-cohesin encounters by their position along the template DNA lent support for this interpretation. Replisome-cohesin encounters in the first third of the template resulted in sister chromatid cohesion only around half of the time. This fraction increased during encounters in the second third, while almost all replisome-cohesin encounters in the final third resulted in stable sister chromatid cohesion (Figure S7A). An alternative scenario that can explain passage events without cohesion establishment is cohesin transfer onto the lagging-strand product only.

### The replisome facilitates cohesin passage

The SYTOX Orange intensity ahead of cohesin transiently increased upon replisome-cohesin encounter in around a third of passage events (12 out of 37), before the replisome passed cohesin (Figure 5B). As seen above in our CMG-cohesin experiments, this observation is suggestive of DNA tension buildup before release upon replisome-cohesin passage.

To quantify how much force the replisome exerts on cohesin before passage, we turned to replisome encounters with immobilized cohesin, tethered to the flow cell surface using the V5 antibody. During encounters with immobilized cohesin, the replisome passage frequency increased to more than half of all events, with successful cohesion establishment in 19 out of 27 passage events (Figures 5D and S6B). Replisomes containing fluorophore-labeled CMG allowed us to measure replisome jump sizes upon cohesin encounter. DNA tension forces that we derived from the jump sizes, as in the case of CMG-cohesin encounters, remained well below 20 pN (Figure 5D). Compared with CMG-cohesin encounters, replisome stall times were briefer, and the jump sizes were shorter (Figure 5E). Therefore, replisome components additional to CMG appear to facilitate cohesin passage.

### Molecular determinants of replisome passage

**Figure 6:**
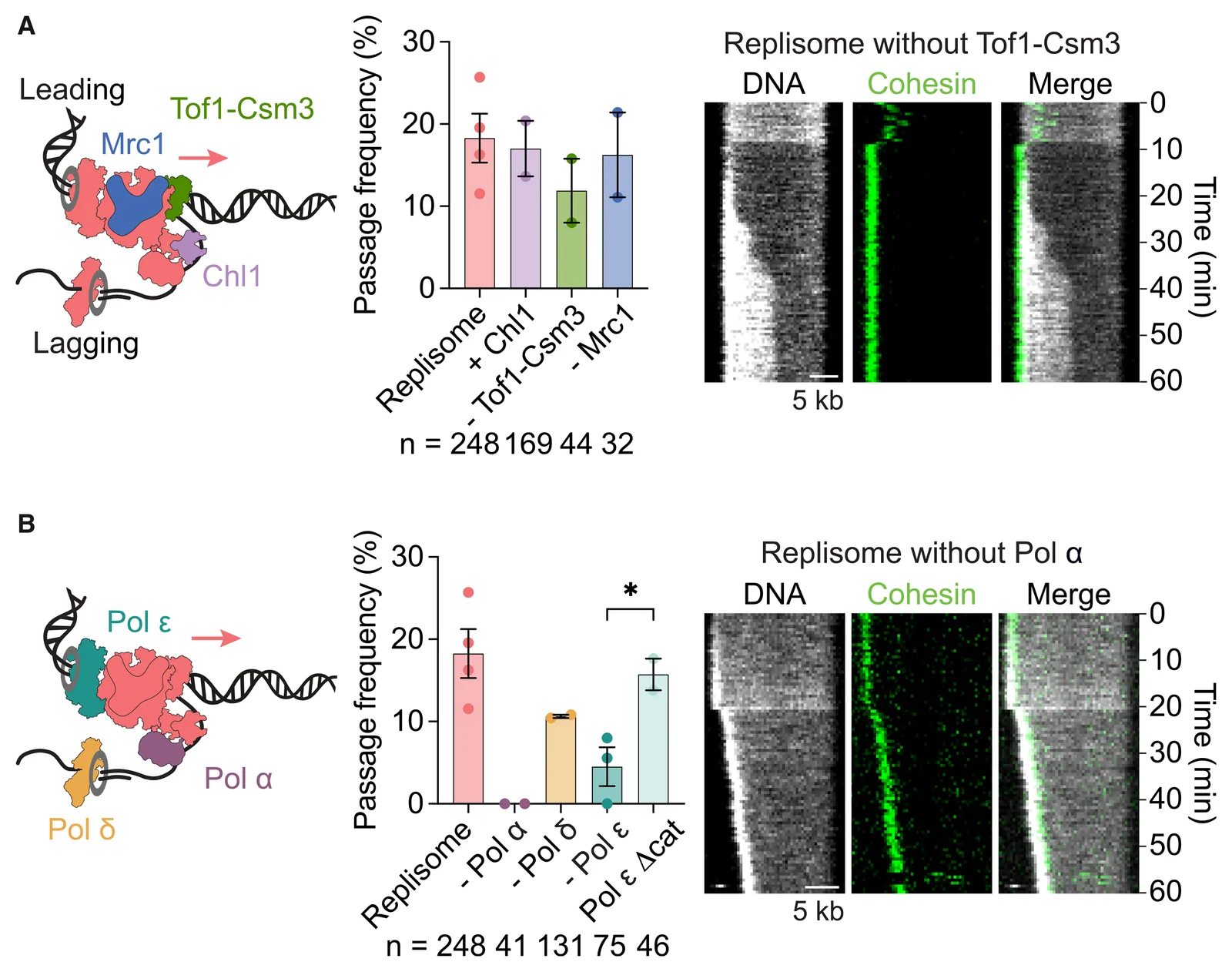
Pol *α* and Pol ε as cohesion establishment factors (A) Schematic depicting known cohesion establishment factors Chl1, Tof1-Csm3, and Mrc1 at the replisome. Quantification of replisome-cohesin passage frequency in the presence or absence of these cohesion establishment factors. *n* is the total number of observed encounters (*p*(replisome vs. -Tof1-Csm3) = 0.2762, unpaired t test). A representative kymograph of a replisome lacking Tof1-Csm3 passing cohesin and establishing sister chromatid cohesion is shown. (B) Schematic depicting the three DNA polymerases Pol α, Pol δ, and Pol ε at the replisome. Quantification of replisome-cohesin passage frequency in the absence of the respective polymerases, or with Pol ε Δcat replacing Pol ε. *n* is the total number of observed encounters (*p*(Pol ε vs. Pol ε Δcat) = 0.0451, unpaired ttest). A representative kymograph of a replisome lacking Pol α encountering cohesin is shown. See also Figure S5 for replication rates and Figure S6 for sister chromatid cohesion establishment frequencies of the various variant replisomes.

We next looked for molecular determinants of a fully assembled replisome that, despite its substantially greater size, help it traverse the cohesin ring more efficiently than CMG alone. We initially investigated the known cohesion establishment factors. To our surprise, the presence or absence of Chl1, Tof1-Csm3, or Mrc1 did not significantly alter the replisome-cohesin passage frequency (Figure 6A). Absence of these cohesion establishment factors also did not alter the efficiency with which replisome-cohesin passage events resulted in the establishment of sister chromatid cohesion (Figure S6B). The positive effect of these cohesion establishment factors on CMG-cohesin passage, observed above, appeared to be supplanted in the context of a full replisome. We will return to the molecular roles of cohesion establishment factors in the discussion section.

In the search for other replisome components that facilitate cohesin passage, we repeated replisome-cohesin encounter assays in the absence of DNA polymerases α, δ, or ε. While all three DNA polymerases are essential for *in vivo* chromosome replication, we can perform *in vitro* DNA replication while omitting one at a time. Removal of Pol δ results in defective lagging-strand DNA synthesis, while only modestly impacting on replisome progression and leading-strand DNA synthesis (Figure S5B).^48^ The frequency of replisome-cohesin passage was somewhat reduced in the absence of Pol δ, though the reduction did not reach statistical significance (Figure 6B), and sister chromatid cohesion was successfully established during the observed cohesin passage events (Figure S6B).

Omission of Pol ε results in Pol δ taking over leading-strand DNA synthesis, albeit at a reduced rate (Figure S5B).^48^ Replication without Pol ε resulted in markedly and significantly reduced replisome-cohesin passage (Figure 6B). Wild-type passage frequency could be restored by adding back a truncated Pol ε variant lacking its N-terminal catalytic domain (Pol ε Δcat), which is incorporated into the replisome but is incapable of DNA synthesis. This finding indicates a role of the non-catalytic Pol ε C-terminal domain in facilitating replisome-cohesin passage. This observation is in line with a sister chromatid cohesion defect that has been observed upon mutation of the Pol ε C terminus.^49^ The role of the Pol ε C terminus in replisome-cohesin encounters remains to be explored.

In the absence of Pol α, leading-strand synthesis initiates from the DNA primer provided on our forked DNA substrate and progresses at close to wild-type rates (Figure S5B).^48^ Lagging-strand priming and synthesis, on the other hand, are abrogated without Pol α. The resultant DNA synthesis pattern is manifest by SYTOX Orange intensity loss behind the replisome, where the single-stranded lagging-strand template is left behind. The leading-strand signal in turn appears as usual while it moves along the template DNA (Figure 6B). Strikingly, replisomes lacking Pol α never passed cohesin (*n* = 41). All recorded replisome-cohesin encounters resulted in cohesin moving together with the replisome, i.e., cohesin was either being pushed along the template or transferred onto the leading-strand DNA product. This observation reveals an integral role of Pol α in replisome passage through the cohesin ring. A Pri1 primase subunit mutation was previously reported to cause a sister chromatid cohesion defect.^50^ The Pol α primase-polymerase complex, bound to CMG (or Ctf4),^51^ might engage with cohesin during replisome passage. Alternatively, the lagging-strand DNA product promotes cohesin ring traversal in an as yet unknown way.

### Replisome and cohesin dynamics during cohesion establishment

**Figure 7:**
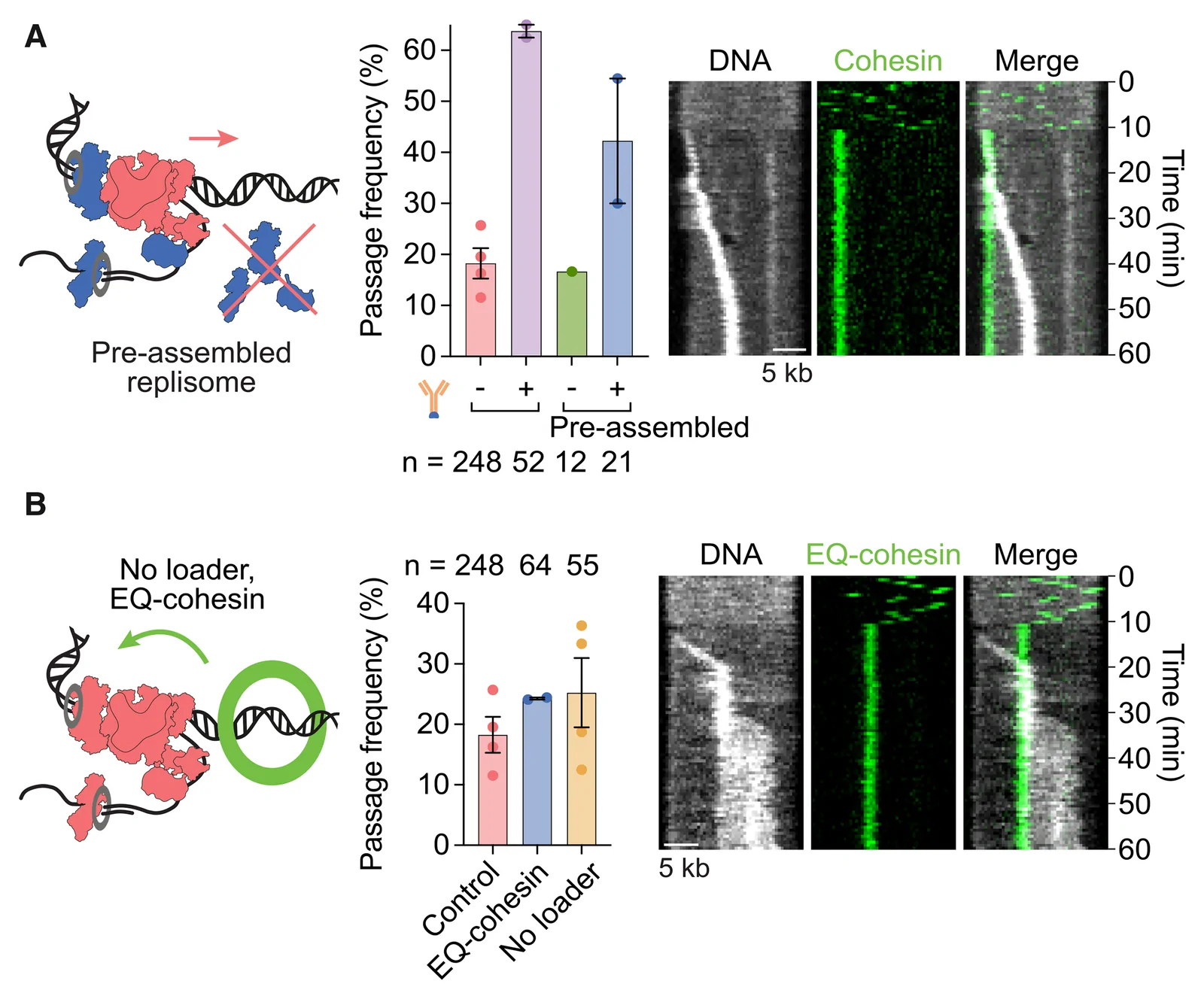
Replisome and cohesin dynamics during cohesion establishment (A) Schematic of a “pre-assembled” replisome, formed on the forked DNA substrate prior to replication initiation in the absence of any free DNA polymerases. Quantification of replisome passage frequencies of mobile and immobilized cohesin. *n* is the total number of observed encounters. A representative kymograph of a preassembled replisome passing cohesin and establishing transient sister chromatid cohesion is shown. (B) Schematic of replisome-cohesin passage in the absence of the cohesin loader or using EQ cohesin. Quantification of replisome-cohesin passage frequency in both cases as well as a representative kymograph of a replisome passing EQ cohesin and establishing sister chromatid cohesion are shown. See also Figure S5 for replisome progression rates, Figure S6 for sister chromatid cohesion establishment frequencies, and Figure S7 for characterization of topological DNA loading of EQ cohesin.

Replicative DNA polymerases dynamically exchange during ongoing DNA replication between the replisome and free soluble enzyme pools.^41^ This behavior raises the question of whether dynamic polymerase turnover forms part of replisome passage through cohesin rings, i.e., DNA polymerases might not in fact be part of the replisome when it passes cohesin. To address this possibility, we performed replisome-cohesin encounter experiments using “pre-assembled” replisomes, in the absence of any free polymerases. We assembled replisomes on our primed fork substrate in the presence of two of the four dNTPs (dATP and dCTP), leading to replisome stalling shortly after assembly. We then washed away any unbound CMG and DNA polymerases before resuming DNA replication by supplementing the two missing dNTPs. The pre-assembled replisomes now traverse the full length of the template without DNA polymerase exchange.^41^ Pre-assembled replisomes progressed at somewhat reduced rates (Figure S5B), but they passed cohesin rings (Figure 7A) and established sister chromatid cohesion with unaltered efficiencies (Figure S6B). These results demonstrate that an intact replisome can pass cohesin rings without a requirement for polymerase dissociation and reassociation.

Finally, we investigated whether cohesin’s enzymatic activity is required during replisome passage. Cohesin possesses ATPase activity that is stimulated by the Scc2–Scc4 cohesin loader. We previously described EQ cohesin with mutations in the Walker B motifs of both the Smc1 and Smc3 ATPase heads. EQ cohesin binds to ATP but hydrolyzes it at a ~ 20-fold reduced rate compared with wild-type cohesin (Figure S5A).^38^ Cohesin initially engages with DNA by forming an ATP-bound DNA-gripping state.^15^ DNA has topologically entered the cohesin ring in this state, but a composite DNA binding surface formed by the cohesin loader and the engaged ATPase heads tightly grips onto the DNA. Consistent with gripping state formation, EQ cohesin was immobile and decorated the DNA template following our usual loading and washing protocol (Figure S7B). We then extended the duration of the washing incubations and included an additional wash at increased stringency. Eventually, individual EQ-cohesin rings remained on DNA that showed diffusive motion,typical of cohesins that have topologically entrapped the DNA. Residual ATP hydrolysis by EQ cohesin, or high-salt-induced disassembly of the gripping state, might have resulted in some cohesins reaching purely topological DNA entrapment.

We now initiated DNA replication and found that replisome passage through EQ cohesin (Figure 7B) and sister chromatid cohesion establishment (Figure S6B) proceeded similarly efficiently as in the case of wild-type cohesin. This observation suggests that ATP hydrolysis by cohesin is not a limiting factor and is possibly unnecessary for cohesion establishment by replisome passage through cohesin rings. Similarly, omitting Scc2-Scc4 had no measurable impact on replisome traversal past cohesin rings and establishment of sister chromatid cohesion (Figures 7B and S6B). Taken together, these findings indicate that neither cohesin’s ATPase activity nor its loader complex is rate limiting for cohesion establishment in this setting, consistent with the possibility that replisomes pass through inert cohesin rings.

## Discussion

In this study, we visualize how a biochemically reconstituted eukaryotic replisome encounters cohesin rings that topologically embrace the template DNA. These experiments illuminate a key moment during the replication and propagation of eukaryotic genomes, the time when two newly synthesized sister genomes are paired by cohesin.

### Replisome-cohesin encounters

Cohesin is thought to exist in a “collapsed” conformational state when bound to DNA.^12–14^ On the other hand, much of the cohesin ring circumference is made up of flexible stretches of coiled coil without a fixed conformation.^9^ High-speed atomic force microscopic visualization of the related condensin complex exemplifies the vast conformational space that SMC complexes sample.^52^ We therefore do not envision cohesin in a defined collapsed state, but rather as a flexible and malleable ring. If approached from one side on DNA, even by an object smaller than its nominal diameter, such a “floppy” ring is displaced. On the other hand, if prevented from escape, an approaching object will unfold the ring. Unfolding might go more or less smoothly, depending on the orientation and conformation in which the replisome finds cohesin. If passage does not readily ensue, force will build up and aid unfolding. These considerations can explain why individual passage events are accompanied by no, or by different degrees of, replisome stalling and force buildup.

While we used an antibody to restrict cohesin’s movement, in a cellular setting, cohesin’s diffusive motion is curtailed by nucleosomes.^12–14^ During chromatin replication, nucleosomes are disassembled from the template DNA only once histones come into direct contact with the replisome.^53^ If cohesin finds itself between a nucleosome and an oncoming replisome, the only place for cohesin to go is the replisome. There, a series of direct protein interactions might facilitate cohesin’s onward transfer. Histones are themselves transferred along the replisome, making use of their own replisome contacts.^53–55^ The resultant histone flow might additionally contribute to moving cohesin along the replisome. The interplay between histone inheritance and cohesion establishment is clearly an area for further exploration.

### Cohesion establishment factors

A key conclusion from our work is that the replisome does not act as a passive cohesin impediment, but it actively facilitates cohesin passage. The more replisome components we added to the CMG helicase, the more efficiently it passed cohesin rings, despite the increasing size. Direct protein interactions between Tof1, Mrc1, Chl1, and cohesin^30,31^ likely form part of a chaperoning mechanism that facilitates passage. On the other hand, in the context of a full replisome, the role of these cohesion establishment factors was surpassed in importance by DNA polymerase α, as well as the non-catalytic C-terminal domain of Pol ε. While we do not know of direct protein interactions between these polymerases and cohesin, Pol α is situated toward the front of the replisome,^51^ while the non-catalytic C-terminal domain of Pol ε is facing rear.^11^

Two other recent studies also found little evidence for Tof1 or Mrc1 contributions to *in vitro* cohesion establishment when using a bare DNA substrate.^31,56^ Could these cohesion establishment factors play their main roles during replication of chromatin? Both Tof1 and Mrc1 orchestrate histone inheritance and make direct histone contacts.^53,54^ It is conceivable that they coordinate histone and cohesin transfer, a role that we could not have observed in our experiments.

Chl1, together with Ctf4, is another cohesion establishment factor that made little contribution to *in vitro* cohesion establishment but performs an important *in vivo* role.^25,56^ Chl1 was recently reported to facilitate topological cohesin loading in the context of DNA replication.^31^ Such a role would again have been missed in our experimental setup, where we topologically loaded cohesin onto the template before initiation of DNA synthesis.

There is much to be learned about cohesion establishment factors, which we now suggest firmly include DNA polymerases α and ε.^49,50^ We are reminded that genetics is a great discovery tool but poor at explaining mechanisms. Now that we have tools to explore the molecular function of each cohesion establishment factor, previously held ideas about their roles might need to be revisited.^3,28,29^ The molecular insight gained will eventually explain their complex genetic relationships.

### Parallel cohesion establishment pathways

Once cohesin is loaded onto DNA, cohesion establishment by replisome passage requires no further ATP hydrolysis by cohesin nor help from the Scc2-Scc4 cohesin loader. The most parsimonious explanation for these observations is that the cohesin ring did not open during passage. This scenario portrays cohesion establishment as a physical process in which the replisome navigates cohesin as an inert, yet flexible, ring.

In a parallel bulk biochemical approach,^56^ we similarly found that *in vitro* cohesion establishment is possible without the cohesin loader. On the other hand, the cohesin loader clearly and substantially contributes to *in vivo* cohesion establishment.^7,57^ An explanation for both findings could derive from the observation that cohesin does not always end up embracing both DNA products during *in vitro* cohesion establishment. In the bulk assays, more often than not, cohesin encircled only one of the two replicated DNAs.^56^ Our single-molecule observations also contained a sizable category of events where cohesin co-migrated with the replisome, maybe because cohesin was transferred to only the leading strand. In other instances, the replisome passed cohesin, but cohesion was not established, and cohesin might have been transferred to only the lagging strand. These observations suggest that replisome passage through the cohesin ring does not always succeed. Might the cohesin ring snap and end up embracing only one of the two sisters? From there, a second DNA capture event that depends on the Scc2-Scc4 cohesin loader^7^ could complete sister chromatid cohesion establishment.

All SMC complexes studied so far share the ability to sequentially, topologically entrap two DNAs,^58,59^ an activity that might be the defining feature of these genome architects. To establish sister chromatid cohesion, cohesin evolved the ability to be acetylated in a replication-coupled reaction^37^ to make otherwise dynamic SMC links permanent. Cohesin evolved an even more startling feature: interactions with replisome components that, if all goes well, allow the replisome to pass through the ring.

### Limitations of the study

We have studied sister chromatid cohesion establishment using a bare DNA substrate, while the presence of histones during chromatin replication will add a layer of complexity to cohesion establishment reactions. Future studies using chromatinized templates should open new ways to probe the contribution of cohesion establishment factors. In cases when the replisome travelled together with cohesin, our single-molecule approach was unable to distinguish whether cohesin was pushed ahead of the replisome or transferred onto the leading strand. Future experiments with altered substrate geometries or added liquid flow might help to differentiate between the two outcomes. Finally, our bulk biochemical experiments revealed an additional cohesion establishment mechanism that uses newly recruited cohesin,^56^ a reaction that remains to be investigated in our single-molecule setup. Given the vital role of sister chromatid cohesion establishment for genome stability, it is unsurprising that there is more than one way to achieve cohesion establishment. Devising methodologies to investigate which establishment pathways are operational *in vivo*, maybe in context-specific manners, remains another important task ahead.

## Resource Availability

### Lead contact

Further information and requests for resources and reagents should be directed to and will be fulfilled by the lead contact, Frank Uhlmann (frank.uhlmann@crick.ac.uk).

### Materials availability

All unique reagents generated in this study will be made available upon reasonable request without restrictions.

### Data and code availability

- All data reported in this paper will be shared by the lead contact upon request.
- This paper does not report original code.
- Any additional information required to reanalyze the data reported in this paper is available from the lead contact upon request.

## Star★Methods

### Key Resources Table

**Table:** 

REAGENT or RESOURCE	SOURCE	IDENTIFIER
**Antibodies**		
Mouse monoclonal anti-V5(Pk)	Bio-Rad	Cat# MCA1360
**Chemicals, peptides, and recombinant proteins**		
SIGMA FAST (protease inhibitor cocktail)	Sigma Aldrich	Cat# S8820
Benzonase Nuclease	Sigma Aldrich	Cat# E1014-5KU
Anti-FLAG M2 affinity gel	Sigma Aldrich	Cat# A2220-25ML
Econo-Pac Chromatography Columns	Bio-Rad	Cat# 7321010
ATP	ThermoFisher	Cat# R0441
TCEP	Fluorochem Limited	Cat# M02624
Pefabloc SC	Roche	Cat# 11429876001
3xFLAG peptide	Custom synthesis	N/A
Calmodulin affinity resin	Agilent	Cat# 214303
MonoQ PC 1.6/5	Cytiva	Cat# 17-0671-01
LD555-maleimide	Lumidyne Technologies	Cat# 4
LD655-maleimide	Lumidyne Technologies	Cat# 8
LD555-NHS	Lumidyne Technologies	Cat# 2
LD655-NHS	Lumidyne Technologies	Cat# 6
Coenzyme A trilithium salt	Sigma Aldrich	Cat# C3019
BG-NH2	New England Biolabs	Cat# S9148S
Superose 6 Increase 3.2/300	Cytiva	Cat# 29-0915-98
Superose 6 Increase 10/300 GL	Cytiva	Cat# 29-0915-96
HisTrap HP column	Cytiva	Cat# 29-0510-21
Biotin-PEG-SVA, MW 20000	Laysan Bio	Cat# Biotin-PEG-SVA-20K-1g
UltraPure BSA	ThermoFisher	Cat# AM2618
Novex™ TBE Gels, 10%, 15W	ThermoFisher	Cat# EC62755BOX
BstXI	New England Biolabs	Cat# R0113L
Sepharose CL-4B	Cytiva	Cat# 17015001
T4 DNA ligase	New England Biolabs	Cat# M0202L
ATP solution	Jena Bioscience	Cat# NU-1010
3-Aminopropyl)triethoxysilane (APTES)	ThermoFisher	Cat# A10668
Biotin-PEG and MPEG (MW 5,000) bundle	Laysan Bio	Cat# BIO-PEG-SVA-5K-100MG & MPEG-SVA-5K-1g
Chloroquine diphosphate crystalline	Sigma Aldrich	Cat# C6628
NeutrAvidin	ThermoFisher	Cat# 31000
SYTOX™ Orange Nucleic Acid Stain	ThermoFisher	Cat# 11368
YOYO-1 Iodide Nucleic Acid Stain	ThermoFisher	Cat# Y3601
XhoI	New England Biolabs	Cat# R0146L
ATPyS	Jena Bioscience	Cat# NU-406-5
AMP-PNP	Jena Bioscience	Cat# NU-407-50
V5 peptide	Custom synthesis	N/A
dNTP bundle	Jena Bioscience	Cat# NU-1005L
NTP bundle	Jena Bioscience	Cat# NU-1014L
6-hydroxy-2,5,7,8-tetramethylchroman- 2-carboxylic acid (Trolox)	Merck	Cat# 648471
Protocatechuic acid (PCA)	Sigma Aldrich	Cat# 03930590
Glucose Oxidase from *Aspergillus niger*	Sigma Aldrich	Cat# G2133-50KU
Catalase from bovine liver	Sigma Aldrich	Cat# C40
**Experimental models: Organisms/strains**		
T7 Express *lysY* Competent *E.coli*	New England Biolabs	Cat# C3010l
All yeast strains used in this study are listed in Table S1.	N/A	N/A
**Oligonucleotides**		
All oligonucleotide sequences used in this study are listed in Table S2.	N/A	N/A
**Recombinant DNA**		
pUber	Lewis et al.^41^	N/A
**Software and algorithms**		
Matlab v.2023b	MathWorks	https://uk.mathworks.com/products/matlab.html
ImageJ/Fiji Version 1.54f	Schindelin, J. et al.^60^	https://imagej.net/software/fiji/
TrackMate v7.13.2	Ershov, D. et al.^61^	N/A
msdanalyzer	Tarantino, N. et al.^62^	https://tinevez.github.io/msdanalyzer/
GraphPad Prism v.10.3.0	Dotmatics	https://www.graphpad.com/
Mars	Huisjes, N. M. et al.^63^	https://duderstadt-lab.github.io/mars-docs/

### Method Details

#### Protein purification and labeling

##### CMG

A *S. cerevisiae* strain overexpressing the 11 CMG subunits (Y6375, strain genotype details can be found in Table S1) was grown overnight at 30°C in YPA (YP with additional 4 mg/L adenine) + 2% raffinose. Cdc45 contained an internal insertion into an unstructured loop, following amino acid 197, of two FLAG epitopes,^64^ plus the 12 amino acid S6 sequence (GDSLSWLLRLLN). The next day, the pre-culture was expanded to 12 L at an optical density OD_600_ = 0.5. When OD_600_ reached ~1.0, protein expression was induced by the addition of 2% galactose (v/v) for 6 h at 30°C. Cells were harvested, washed with water and resuspended in CMG buffer (25 mM HEPES-NaOH pH 7.5, 300 mM KCl, 2 mM Mg(OAc)_2_, 0.02% Tween-20, 0.5 mM TCEP, 10% glycerol) supplemented with protease inhibitors (SigmaFAST, Sigma-Aldrich). This cell suspension was frozen dropwise in liquid nitrogen, crushed by freezer milling and the cell powder was stored at -80°C.

The cell powder was thawed and resuspended in CMG buffer supplemented with protease inhibitors, 0.5 mM AEBSF, and 3000 Units benzonase for 12 L starting culture (Sigma-Aldrich). After stirring for 30 min at 4°C, cell debris was removed by ultra-centrifugation in a Ti45 rotor at 40,000 rpm for 60 min. The clarified lysate was transferred onto anti-FLAG M2 agarose beads (Sigma-Aldrich, 4 mL of beads for 12L of culture) equilibrated in CMG buffer and incubated on a rocking platform at 4°C for 4 h. Flag beads were transferred into two 20 ml disposable columns (BioRad), washed with 50 column volumes (cv) CMG buffer and then incubated with ATP wash buffer (CMG buffer with added 1 mM ATP) for 10 min. After further 15 cv CMG buffer wash, proteins were eluted by first incubating for 40 min in 2 cv of CMG buffer supplemented with 0.5 mg/ml 3xFLAG peptide, followed by 20 min in 1 cv of CMG buffer supplemented with 0.25 mg/ml 3xFLAG peptide. The eluted fractions were combined, supplemented with 2 mM CaCl_2_, and incubated overnight at 4°C with calmodulin affinity resin (2 mL resin for 12L of culture). The resin was collected in a 20 mL disposable column, washed with 50 cv calmodulin wash buffer (CMG buffer with 2 mM CaCl_2_), then proteins were eluted with calmodulin elution buffer (CMG buffer with 2 mM EDTA and 2 mM EGTA). The calmodulin eluate was loaded onto a MonoQ PC 1.6/5 column (Cytiva) and separated with a 300-600 mM KCl gradient over 10 cv. Peak fractions were pooled, snap frozen in liquid nitrogen and stored at -80°C.

For fluorophore labelling, CMG in the pooled MonoQ fractions was incubated with phosphopantetheinyl transferase^65^ and LD655-CoA (Lumidyne Technologies, 1:2:5 molar ratio) in the presence of 10 mM MgCl_2_ at 23°C for 1 hour. The labelling reaction was centrifuged for 10 min at 13,000 rpm in a benchtop centrifuge at 4°C followed by removal of phosphopantetheinyl transferase and excess dye using Superose 6 Increase 3.2/300 (Cytiva) chromatography in CMG buffer. Peak fractions were pooled, snap frozen in liquid nitrogen and stored at -80°C.

##### Cohesin-SNAP and EQ-cohesin-SNAP

A tetramer *S. cerevisiae* cohesin-SNAP complex was expressed and purified as previously described.^38^ The SNAP-tag was fused to the C-terminus of Smc3. Following the heparin chromatography step, the pooled peak fractions were incubated with benzylguanine-LD555 or benzylguanine-LD655 (Lumidyne Technologies) in a protein-to-dye ratio of 1:2 in the presence of 1 mM DTT overnight at 4°C. Excess dye was removed by gel filtration on a Superose 6 Increase 10/300 GL column (Cytiva) equilibrated in gel filtration buffer (50 mM Tris-HCl pH 7.5, 150 mM NaCl, 0.5 mM TCEP, 10% glycerol). The peak fractions were pooled, snap frozen in liquid nitrogen and stored at -80°C.

##### Scc2-Scc4 and Chl1

The *S. cerevisiae* cohesin loader Scc2-Scc4 complex and Chl1 were expressed and purified as previously described.^30,38^

##### Protocatechuate 3,4-Dioxygenase (PCD)

PCD from *Pseudomonas putida* was expressed and purified as previously described with minor changes.^66^ T7 express lysY cells (New England Biolabs) carrying a PCD expression vector (pVP91A-pcaHG, Addgene) were grown at 37°C overnight in LB broth supplemented with carbenicillin. In the following morning, cells were diluted 1:100 into 2 × 1L medium and further incubated at 37°C until an OD_600_ of 0.5 was reached. Cells were transferred to 17°C, and once an OD_600_ of 0.7 was reached, the expression of PCD was induced by addition of 0.5 mM IPTG and 10 mg/L Fe(NH_4_)_2_(SO_4_)_2_. The cells were maintained at 17°C for ~18 h. Next, cells were harvested, resuspended in PCD buffer (50 mM Tris-HCl pH 7.5, 500 mM NaCl, 10 mM Imidazole, 10% glycerol) supplemented with protease inhibitors and flash frozen in liquid nitrogen. Cells were lysed by subjecting them to three consecutive freeze-thawing cycles followed by sonication. Cell debris was removed by centrifugation, and the clarified lysate was applied to a HisTrap (Cytiva) column that had been equilibrated in PCD buffer. The column was washed with PCD buffer containing 20 mM imidazole, followed by elution with two consecutive 15 cv steps of increasing imidazole concentrations (125 mM and 250 mM imidazole, respectively). The peak fractions were pooled and concentrated using centrifugal filters with a 10 kDa molecular weight cut-off (Merck). Finally, the concentrated protein was applied to a Superdex 200 Increase 10/300 size exclusion column (Cytiva) equilibrated in buffer containing 50 mM Tris-HCl pH 7.5, 100 mM NaCl, 0.1 mM EDTA, 10% glycerol. Peak fractions were pooled, flash frozen in liquid nitrogen and stored at -80°C. The activity of purified PCD was measured using an enzymatic activity assay, whereby the decrease in protocatechuic acid (Sigma-Aldrich) was monitored over time as absorbance decrease at 290 nm.

##### Replication proteins

Additional DNA replication factors, Mcm10, Tof1-Csm3, Mrc1, RPA, PCNA, RFC, Ctf4, Pol α, Pol δ, and Pol ε were expressed and purified as previously described.^48,64,67^

##### V5-antibody functionalization

V5-antibody (Bio-Rad) was functionalised with a 20 kDa biotin-PEG-SVA linker (Laysan Bio) through NHS-ester coupling. Biotin-PEG-SVA was dissolved in DMSO to a final concentration of 3 mM. The V5-antibody and the dissolved PEG were mixed in a 1:20 ratio and incubated for two hours at 22°C and agitated at 400 rpm. The reaction was quenched by the addition of 2 μl of 1 M TrisHCl pH 7.5. The reaction was applied to a Superose 6 Increase 10/300 GL size exclusion column which had been equilibrated in PBS. Three peaks were observed during elution and, to ascertain the number of PEG labels per antibody, peak fractions were analysed by mass photometry (Figure S3A). The single-PEG labelled antibody fractions were pooled, snap frozen in liquid nitrogen and stored at -80°C.

#### In vitro *ensemble assays*

##### Cohesin ATPase and DNA loading assays

The rate of ATP hydrolysis by cohesin and its ability to load onto DNA in a salt-resistant manner were determined using previously established assays.^2,38^

##### CMG helicase assay

CMG unwinding was determined following a previously published protocol with minor changes.^42^ The forked DNA substrate was assembled by annealing 50duplex-lag-FAM and 50duplex-lead in TE (oligonucleotide sequences can be found in Table S2). The mix was heated to 95°C and slowly cooled to 20°C. The assembled fork was purified by TBE gel electrophoresis, electroelution and dialysis against storage buffer (20 mM Tris-HCl pH 8.0, 20 mM NaCl). The fluorescent forked DNA concentration was determined using the fluorescently labelled oligonucleotide as the standard.

For CMG unwinding assays, 10 nM CMG and 3 nM forked DNA were mixed in CMG buffer (25 mM Tris-HCl pH 7.5, 10 mM MgAc_2_, 250 mM potassium glutamate, 0.0025 % Tween 20, 1 mM TCEP, 0.1 mg/mL BSA) on ice. The reaction was started by addition of 3 mM ATP and incubated at 30°C. Aliquots were retrieved at indicated times and quenched by addition to 3xStop buffer (30 mM Tris-HCl pH 7.5, 60 mM EDTA, 1.5% SDS, 15% Sucrose) and separated on a 10% acrylamide TBE gel. The gel was imaged using a fluorescence imager and the unwinding activity was quantified as the amount of unwound 50duplex-lag-FAM using ImageJ.^60^

##### Linear forked DNA substrate for single molecule experiments

The linear forked DNA substrate was prepared following previously published protocols with minor changes.^41,68^ First, pUber was digest with BstXI (2.5 Units per μg of DNA, New England Biolabs) in NEBuffer 3.1 for 8 hours at 37°C. The reaction was quenched by addition of EDTA (12 mM final) and NaCl (300 mM final), and digested DNA was separated by size exclusion chromatography over a home-made Sepharose CL-4B chromatography column equilibrated in eluent buffer (10 mM Tris-HCl pH 8.0, 12 mM EDTA, 300 mM NaCl). Peak fractions were pooled and dialysed two times for 1 hour against TE (10 mM Tris-HCl pH 8.0, 1 mM EDTA). Capping fragments were generated by annealing oligonucleotides in two separate reactions in 1xTE, blockLd-biteg and blockLg were annealed in a 1:6 molar ratio, 99Lg-biteg, 160Ld and Fork-primer were annealed in a 1:6:60 molar ratio. Annealing reactions were heated to 95°C and, after 2 minutes, the temperature was reduced 1°C every 40 sec until reaching 20°C. The annealed capping fragments were now ligated to linearised pUber using T4 DNA ligase (100 Units per μg of DNA, New England Biolabs) in 1xCutSmart supplemented with 1 mM ATP and 1 mM DTT. The ligation reaction was incubated overnight at 16°C and then quenched with 12 mM EDTA and 30 mM NaCl. Excess capping oligos and T4 DNA ligase were removed by size exclusion chromatography over a Sepharose CL-4B chromatography column equilibrated in eluent buffer. Peak fractions were pooled, aliquoted and snap frozen in liquid nitrogen.

#### Flow cell preparation

##### Functionalisation of coverslips

Coverslips used for single-molecule experiments were cleaned, silanised and PEG functionalised based on previous protocols with adjustments.^69–71^ Coverslips (24×60 mm, Marienfeld Superior) were placed into a polypropylene staining jar, filled with anhydrous ethanol and sonicated for ~20 min. The ethanol was removed, and coverslips washed 10 times with purified water. The jar was filled with 5 M KOH and again sonicated for ~20 min. KOH was removed, and coverslips washed 10 times with purified water. The ethanol and KOH washes were repeated. Coverslips were carefully dried with nitrogen gas and then plasma cleaned for 3 min at max intensity in a PDC-002-CE device (Harrick Plasma). For silanisation, coverslips were first washed several times with acetone, followed by incubation in 2% 3-aminopropyltriethoxysilane (Alfa Aesar) in acetone with shaking. The silane solution was decanted, the coverslips washed once with acetone and then sonicated for 30 sec in acetone. Subsequently, coverslips were washed 20 times with purified water and immediately dried with nitrogen gas.

For 6 coverslips, 75 mg mPEG-succinimidyl valerate (SVA) and 3 mg biotin-PEG-SVA (MW 5000, Laysan Bio Inc.) were mixed and dissolved in 500 μl 0.1 M NaHCO_3_ Three of the silanised coverslips were placed face-up in an empty freezer box, humidified by water at the edges. 150 μl of PEG solution was added and the three remaining coverslips placed on top, face-down, sandwiching the PEG solution. After ~3 h, the PEGylated coverslips were rinsed, placed into a clean jar and washed 20 times with purified water. Coverslips were dried with nitrogen gas and the PEGylation process was repeated, this time incubating overnight. Rinsed and dried PEGylated coverslips were stored under vacuum for up to 2 weeks.

##### Flow cell assembly

A standard flow cell comprised 7 flow channels, cut into parafilm and sandwiched between one PEGylated coverslip and a quartz coverglass (cleaned in acetone and sonication for 30 min in ethanol). The coverglass contained 7 corresponding pairs of drilled holes. The assembly was secured by melting the parafilm on a heating block for 3 min at 110°C, then sealing the edges with epoxy glue. PE60 tubing (0.76/1.22 mm, Stoelting) was fit into the coverglass holes and sealed with epoxy glue. For side flow experiments, a flow channel comprised one straight channel with a second channel branching at a right angle from the flow cell centre to a second outlet.

#### Single-molecule assays

All buffers used in single-molecule experiments were filtered and degassed for at least 30 min immediately before use.

##### DNA tethering

Linear forked DNA was tethered to a PEGylated coverslip surface via biotin-streptavidin interaction. An assembled flow cell was placed onto the microscope, with the outlet tubing of one flow channel connected to a microfluidics pump (Harvard Apparatus), and the inlet placed into sample tubes containing the indicated solutions. Washing and DNA tethering steps were performed at a flow rate of 100 μl/min. First, the channel was flushed with 100 μl blocking buffer BB (20 mM Tris-HCl pH 7.5, 50 mM NaCl, 2 mM EDTA, 0.005%Tween-20) including 0.2 ml/ml NeutrAvidin (Thermo Fisher) and incubated for 30 min without flow. Then the flow was restarted and the channel washed with 300 μl BB. Now 450 μl of 10 pM linear forked DNA in BB, supplemented with 0.2 μM chloroquine (Sigma-Aldrich) was introduced, and untethered DNA was immediately flushed out with further BB.

##### Cohesin loading

Prior to cohesin loading, the surface of the DNA containing flow channel was additionally passivated by incubating for 20 min with 1 mg/ml Ultrapure BSA (Thermo Fisher) in cohesin loading buffer CLB (35 mM Tris-HCl pH 7.5, 25 mM NaCl, 25 mM KCl, 1 mM MgCl_2_, 1 mM DTT, 0.003% Tween-20, 5% glycerol). For cohesin loading, 1.5 nM fluorescently labelled cohesin and 3 nM Scc2-Scc4 were introduced into the flow channel at 20 μl/min for 150 μl in CLB supplemented with 1 mM ATP (Jena Bioscience) and 1 mg/ml BSA. Excess and non-topologically loaded cohesin were immediately removed by washing with 100 μl CMG buffer (25 mM Tris-HCl pH 7.5, 250 mM Potassium glutamate, 10 mM Mg(OAc)_2_, 0.0025% Tween-20, 1 mM DTT) supplemented with 150 mM NaCl, followed equilibration in CMG buffer. Cohesin was imaged in CMG buffer supplemented with 150 nM SYTOX Orange or 5 nM YOYO-1 (both Thermo Fisher) and an oxygen scavenging system (see below). Where indicated, the restriction enzyme XhoI (40 units/ml) in CMG buffer supplemented with 150 nM SYTOX Orange, 0.1 mg/ml BSA and oxygen scavenger was introduced into the flow channel at 50 μl/min over 900 μl.

For EQ-cohesin loading, 0.5 nM fluorescently labelled EQ-cohesin and 1 nM Scc2-Scc4 were introduced into the flow channel at 20 μl/min for 150 μl in CLB supplemented with 1 mM ATP and 1 mg/ml BSA. Excess and non-topologically loaded cohesin were immediately removed by extensive washing first with 200 μl CMG buffer supplemented with 150 mM NaCl. We now added an additional wash with 200 μl CMG buffer supplemented with 500 mM NaCl, followed by further 200 μl CMG buffer supplemented with 150 mM NaCl, before final re-equilibration in CMG buffer.

##### CMG loading and translocation

Following BSA passivation as above, 1 nM CMG-LD655 and 10 nM Mcm10 were introduced into the flow channel at 20 μl/min for 100 μl in CLB supplemented with 0.33 mM ATP and 1 mg/ml BSA. Excess CMG was immediately removed by washing with CMG buffer supplemented with 150 mM NaCl, followed by CMG buffer. CMG was activated and translocation was imaged in CMG buffer supplemented with 10 nM Mcm10, 3 mM ATP, 1 mg/ml BSA and oxygen scavenger. Where indicated, CMG was loaded in presence of 0.33 mM ATPγS and 300 nM RPA was included during activation and imaging.

##### CMG-cohesin encounters

Cohesin-LD555 was loaded onto tethered DNA as above. Next, CMG-LD655 was loaded, excess CMG removed, and CMG activated as described. Cohesin-CMG encounters were imaged for 40 min in CMG buffer supplemented with 10 nM Mcm10, 3 mM ATP, 0.1 mg/ml BSA and oxygen scavenger. For experiments with cohesion establishment factors, 30 nM Tof1-Csm3-Mrc1 or Chl1-Ctf4 were added during activation and imaging. The same experimental design was used when imaging converging CMGs.

For experiments with cohesin immobilisation, following loading, cohesin was initially imaged for 5 minutes to identify topologically loaded and freely diffusing complexes. Now, 25 nM V5 PEG-biotin antibody in CMG buffer with 0.1 mg/ml BSA was introduced over 100 μl at 20 μl/min. After incubation for 3 min, the channel was washed with CMG buffer in preparation for CMG loading and activation. For cohesin elution, the flow channel was flushed with CMG buffer supplemented with 50 μg/ml V5 peptide, 0.1 mg/ml BSA and oxygen scavenger, and imaged for a further 25-30 min. The DNA was afterwards stained with 150 nM SYTOX Orange in CMG buffer.

##### DNA replication

For additional passivation, the flow channel was incubated with 2% Tween-20 in BB for 10 min prior to DNA tethering. After DNA attachment and equilibration in CMG buffer, 10 nM unlabelled CMG in CMG buffer supplemented with 0.33 mM AMP-PNP and 0.1 mg/ml BSA was flushed into the flow channel at 20 μl/min and incubated for 10 min. Replication was initiated by introducing 60 nM Mcm10, 20 nM Ctf4, 30 nM Tof1-Csm3-Mrc1, 200 nM RPA, 20 nM PCNA, 20 nM RFC, 20 nM Pol α, 20 nM Pol δ and 20 nM Pol ε in CMG buffer (containing 200 mM potassium glutamate) supplemented with 150 nM SYTOX Orange, 125 μM dNTPs, 250 μM NTPs (both Jena Bioscience), 5 mM ATP and 0.1 mg/ml BSA.

When using pre-assembled replisomes, following CMG loading, replisomes were assembled by introducing 60 nM Mcm10, 30 nM Ctf4, 30 nM Tof1-Csm3-Mrc1, 20 nM PCNA, 20 nM RFC, 20 nM Pol α, 20 nM Pol δ and 20 nM Pol ε in CMG buffer (containing 200 mM potassium glutamate) supplemented with 150 nM SYTOX Orange, 60 μM dCTP/dATP, 0.33 mM ATP and 0.1 mg/ml BSA at 20 μl/min for 100 μl. Replication was initiated by flushing the flow channel with 60 nM Mcm10, 20 nM Ctf4, 30 nM Tof1-Csm3-Mrc1, 200 nM RPA, 20 nM PCNA, 20 nM RFC in CMG buffer (containing 200 mM potassium glutamate) supplemented with 150 nM SYTOX Orange, 125 μM dNTPs, 250 μM NTPs, 5 mM ATP and 0.1 mg/ml BSA.

##### Replisome-cohesin encounters

Replisome-cohesin encounter experiments were carried out following the same workflow as CMG-cohesin encounters with minor adjustments. The surface was passivated with 2% Tween-20 prior to DNA attachment, and all buffers were supplemented with 0.1 mg/ml BSA. Cohesin-LD655 was loaded, washed and imaged as described. Unlabelled CMG was then loaded, and replication was initiated as detailed above, with the additional inclusion of an oxygen scavenging system. For experiments with immobilised cohesin, the immobilisation and V5 peptide elution steps followed the same procedure as described under CMG-cohesin encounters. For experiments that included LD655-labelled CMG, 1 nM CMG introduced and incubated for 5 minutes in the presence of AMP-PNP, before replication proteins and nucleotides were added.

##### Side flow experiments

The protein and buffer conditions in side flow experiments remained unchanged, unless specified otherwise. The side flow channels were prepared as described above, with the main and side outlets connected to separate microfluidics pumps. First, NeutrAvidin in BB was flushed through both channels and incubated for 30 min. Both channels were washed with 200 μl BB and linear forked DNA (20 pM in BB containing 0.2 μM chloroquine) was introduced through the main channel at 50 μl/min over 250 μl. The unbound DNA was washed sequentially through the main and side channels with BB. After incubating both channels with 1 mg/ml BSA in CLB, cohesin was loaded at 20 μl/min over 150 μl through the main channel. After sequential washes of both channels with CMG buffer containing 150 mM NaCl, cohesin was imaged in CMG buffer supplemented with 5 nM YOYO-1, oxygen scavenger, and 0.1 mg/ml BSA. Now, side flow was applied at 50 μl/min and cohesin immobilised with 50 nM V5 PEG-biotin antibody in CMG buffer with 0.1 mg/ml BSA for 6 min. Both channels were washed with CMG buffer and CMG was loaded at 20 μl/min for 100 μl through the main channel. Excess CMG was removed by washing through both channels using CMG buffer containing 150 mM NaCl, followed by CMG buffer. Finally, CMG was activated, and cohesin-CMG encounters were imaged in CMG buffer supplemented with 10 nM Mcm10, 5 nM YOYO-1, 3 mM ATP, 1 mg/ml BSA and oxygen scavenger while maintaining a constant flow of 5 μl/min through the main channel. After 40 min, cohesin was eluted with 50 μg/ml V5-peptide in CMG buffer supplemented with 5 nM YOYO-1, 0.1 mg/ml BSA and oxygen scavenger.

##### Oxygen scavenging systems

Two oxygen scavenging systems were used to increase fluorescence dye stability. The first consisted of PCD (see above), Protocatechuic acid (PCA) and 6-hydroxy-2,5,7,8-tetramethylchroman-2-carboxylic acid (Trolox) (both Sigma-Aldrich), and was used for imaging of cohesin, CMG and cohesin-CMG encounters. CMG imaging buffer was supplemented with 20 nM PCD, 2.5 mM PCA (from a 650 mM stock in DMSO) and 1 mM Trolox (from a 4 mM stock in CMG buffer). The second oxygen scavenging system comprised glucose oxidase, catalase and glucose and was used for cohesin-replisome encounter experiments. 0.2 mg/ml glucose oxidase (from a 20 mg/ml stock in 50 mM Tris-HCl pH 7.5, 50 mM NaCl and 50% glycerol), 0.035 mg/ml catalase (from a 3.5 mg/ml stock in the same buffer) and 0.5% glucose were added to CMG imaging buffer.

#### Data acquisition, processing and analysis

##### Imaging

All experiments were performed on a Nikon Eclipse Ti2-E inverted TIRF microscope equipped with a SR HP Apo TIRF 100x/1.49 oil immersion objective and a LU-NV-D laser bed. The imaging temperature was maintained at 30°C in an electrically heated chamber (Okolab). Cohesin diffusion and photobleaching kinetics were captured every 100 ms for 1-2 min. Other experiments were typically imaged every 20 sec in an area covering 4×4 field of views. The fluorescence signal was spectrally separated using a T635lpxr-UF2 beam splitter (Chroma) and recorded on two Prime 95B sCMOS cameras (Teledyne photometrics) with 0.11 μm x 0.11 μm pixel size. The system was controlled using Nikon NIS-Elements software.

##### Image processing

Fields of view were first separated into individual files and saved in tiff format. Subsequent image processing and analysis were carried out in Fiji (Version 1.54f). Each image was corrected for stage drift using a custom script.^41^ Channels were aligned manually using an overexposed SYTOX Orange stain image as a guide. Individual events occurring on DNA were cropped and saved as individual files for later analysis. Kymographs across a 3 pixels wide line were created using the KymoResliceWide plugin (https://doi.org/10.5281/zenodo.4281086).

##### Tracking of CMG translocation and replication

Single fluorescent spots on DNA were tracked using the Dog detector and Linear-motion LAP tracker in TrackMate (v7.13.2)^61^ and the resulting trajectories were exported to Mars.^63^ Kinetic change point analysis was used to fit individual rate segments^72^ and the obtained rates were exported for further statistical analysis using Matlab (v.2023b) and GraphPad Prism (v.10.3.0). CMG translocation rates were weighted by the duration of a segment.

##### Tracking of cohesin for photobleaching and MSD analyses

Single fluorescent cohesin on DNA was tracked, and trajectories exported to Mars, as above. The mean intensity of cohesin was plotted over time and kinetic change point analysis was used to fit photobleaching steps.^72^ Mean square displacement (MSD) analysis was performed using the Matlab class msdanalyzer.^62^ The diffusion coefficient of individual cohesin molecules was estimated from the slope of a linear fit to the first five time delays (0.1-0.5 s). Only coefficients with a goodness of fit of R^2^ < 0.8 were considered.

##### Force calculations

Forces generated by CMG or replisome-cohesin encounters that led to DNA stretching prior to CMG or replisome jump events were calculated using a force-extension formula for worm-like chain polymers.^46^ DNA tension in the DNA segment in front of CMG (*F*) was calculated using the worm-like chain equation:

$$F=\frac{kT}{L_{P}}\left(\frac{1}{4}\frac{1}{{\left(1-x\right)}^{2}}-\frac{1}{4}+x\right)$$

Where *k* is the Boltzmann constant, *T* the absolute temperature, *L*_*P*_ = 50 nm is the persistence length of DNA, and x is the extension ratio of the DNA, which was obtained as follows:

$$x=\frac{L}{L_{0}}\;\text{and}\;L_{0}=\frac{d}{D}\times L_{c}$$

Here, *L* represents the end-to-end distance of the stretched DNA segment immediately before the jump, and *L*_*0*_ is the contour length of that DNA segment. The contour length was calculated assuming that the jump releases and equilibrates tension in the DNA. Therefore, the contour length of the segment is the ratio between the end-to-end distances of the segment *d* and the total DNA *D*, multiplied by the total DNA contour length. For the DNA used here, the contour length of the total DNA was *L*_*c*_ = 6.22 μm.

The DNA length *D* was obtained using the DNA finder tool in Mars.^63^ The length of the stretched DNA segment before and after the CMG or replisome jumps were determined by first tracking the CMG using TrackMate, followed by combining the CMG track with the DNA location in Mars. This allowed us to determine where on the DNA CMG was before (*L*) and after the jump (*d*).

### Quantification and Statistical Ananlysis

Plots showing individual data points were generated using GraphPad Prism (v.10.3.0). The number of molecules n is indicated in each figure, or its legend. All experiments were conducted in at least two biological replicates. Statistical significance was evaluated using an unpaired *t*-test with statistical significance defined as *p* < 0.05. All error bars are defined in the figure legends and typically represent the standard error of the mean.

## Supplementary Material

Supplemental information can be found online at https://doi.org/10.1016/j.cell.2025.08.028.

Table S1

Supplemental figures

## Highlights

- The replication machinery passes through topologically loaded cohesin rings
- Replisome passage results in sister chromatid cohesion establishment
- Restricting cohesin mobility, mimicking chromatin, promotes passage
- DNA polymerases ***α*** and **ε** are molecular determinants for cohesion establishment

## In brief

Glaser et al. use single-molecule imaging to visualize encounters between the DNA-replication machinery and cohesin rings in real time. They see replisomes passing through cohesin rings—a simple physical act that establishes sister chromatid cohesion, required for faithful genome inheritance.
