# Supplementary material for: Replisome passage through the cohesin ring
Source: Cell. Author manuscript; Available in PMC 2026 Aug 26. (PMC7619401; doi:10.1016/j.cell.2025.08.028)
Supplement: Supplementary file 1 [file EMS217828-supplement-Supplemental_figures.pdf]

CellPress
OPEN ACCESS

Supplemental figures

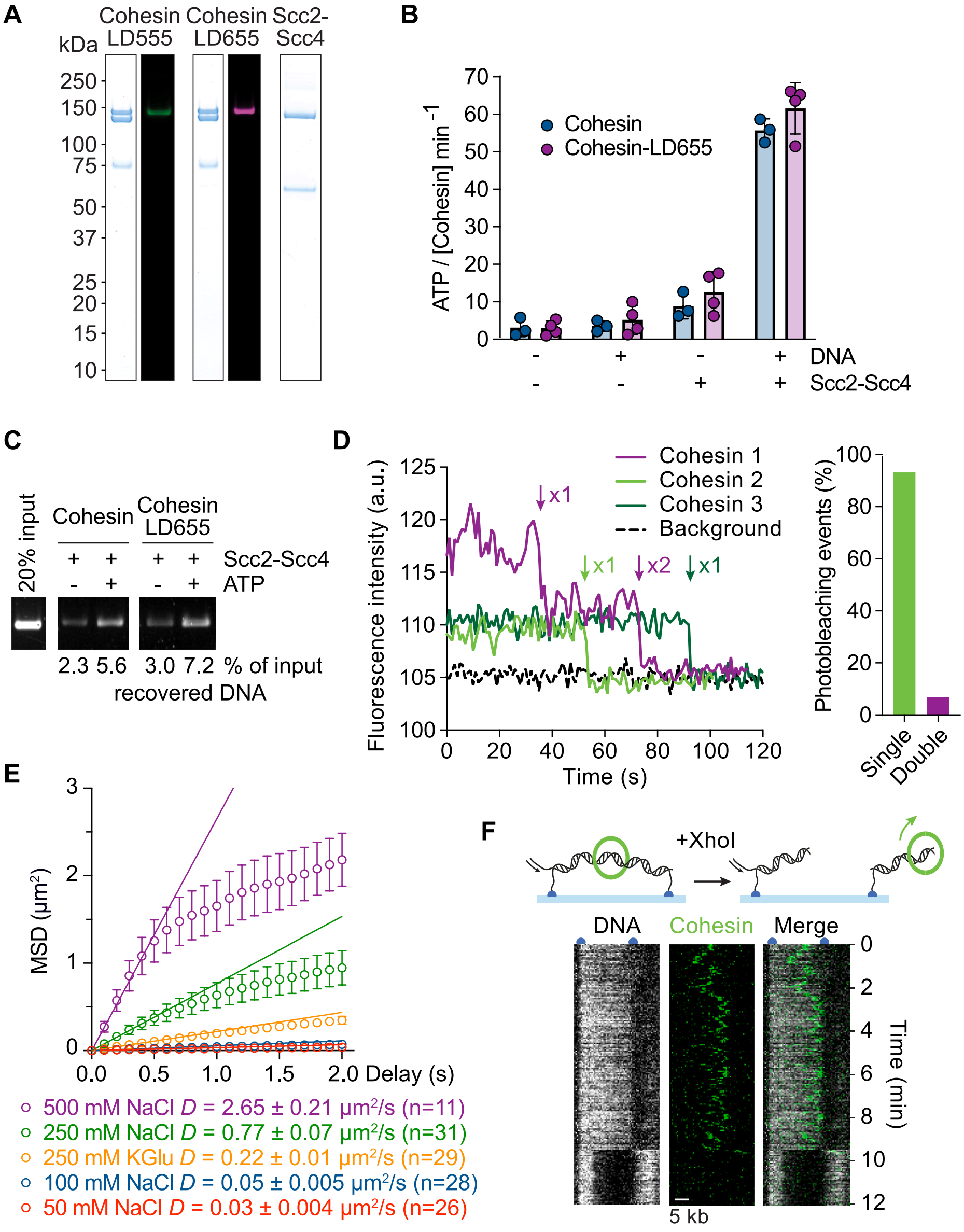

Figure S1. Ensemble and single-molecule analyses of budding yeast cohesin, related to Figure 1

(A) Purified and fluorophore-labeled budding yeast cohesin tetramer complexes (Smc1, Smc3, Scc1, and Scc3) were analyzed by SDS-polyacrylamide gel electrophoresis and Coomassie blue staining or in-gel fluorescence. The purified, unlabeled Scc2–Scc4 cohesin loader complex was analyzed alongside.

(B) ATP hydrolysis rates of unlabeled and LD655-labeled cohesin in presence or absence of DNA and Scc2–Scc4. Each point shows the result from independent biological replicate experiments. Bars show the mean, and error bars show the standard deviation.

(C) Gel image of DNA recovered in a cohesin loading assay with unlabeled and LD655-labeled cohesin, in the presence of Scc2–Scc4, with or without added ATP. The amount of recovered DNA was quantified and compared with the input.

(D) Example photobleaching traces of cohesin on DNA, with single and double bleaching steps highlighted by arrows. The percentage of single- and double-step events is shown ($n = 44$).

(E) Mean square displacement (MSD) as a function of delay time for cohesin on DNA, imaged at increasing salt concentration. The mean and standard error are plotted for each delay time. Data points up to delay times of 0.5 s were fitted with linear regressions, where the slope denotes the diffusion coefficients (D, indicated).

(F) Schematic and representative kymograph of cohesin sliding off the free end of surface-tethered DNA following restriction digestion.

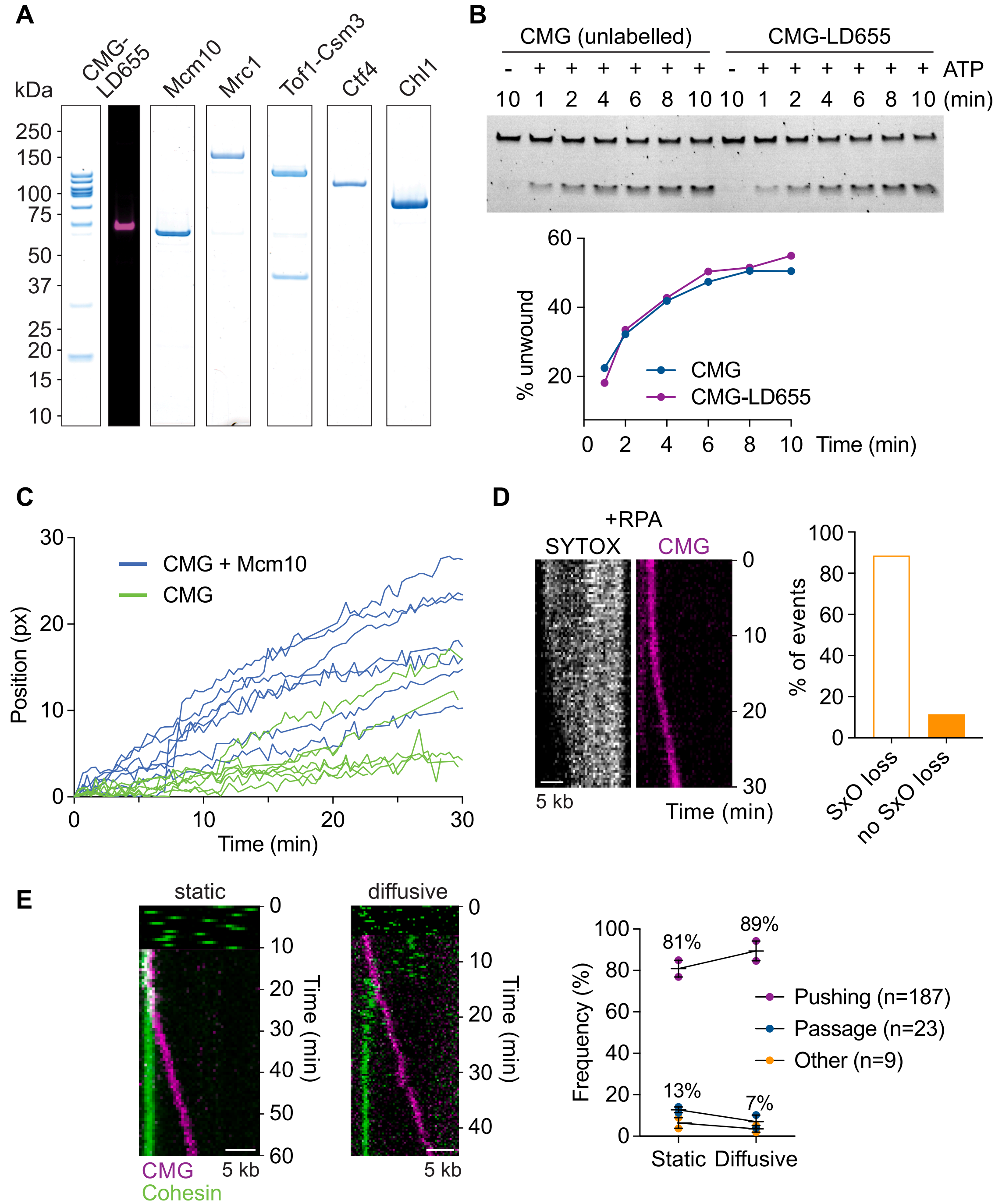

Figure S2. Ensemble and single-molecule analyses of budding yeast CMG, related to Figure 1

(A) Purified and fluorophore-labeled budding yeast CMG was analyzed by SDS-polyacrylamide gel electrophoresis and Coomassie blue staining or in-gel fluorescence. Purified, unlabeled Mcm10, Mrc1, Tof1-Csm3, Ctf4, and Chl1 were analyzed alongside.

(B) CMG unwinding assay comparing unlabeled and labeled CMG. The fluorescence gel image shows the fork substrate and unwound single-stranded product. Quantification of the fraction of unwound DNA over time is shown.

(C) Example trajectories of CMG positions along the DNA as a function of time, in the presence or absence of Mcm10.

(D) Representative kymograph of DNA unwinding by CMG in the presence of RPA. SYTOX Orange preferentially stains dsDNA, with loss of the SYTOX Orange signal indicating DNA unwinding. The percentage of CMG translocation events that were accompanied by SYTOX Orange signal loss is indicated (n = 190).

(E) Representative kymographs of CMG encountering either a static or a diffusive cohesin molecule. In both shown examples, CMG passes cohesin. Quantification of the encounter outcomes is shown (where of all encounters 59% were with static and 41% with diffusive cohesin). The individual results from biological repeat experiments are shown, with the means and standard errors indicated. The total sample size (n) for each outcome category is given.

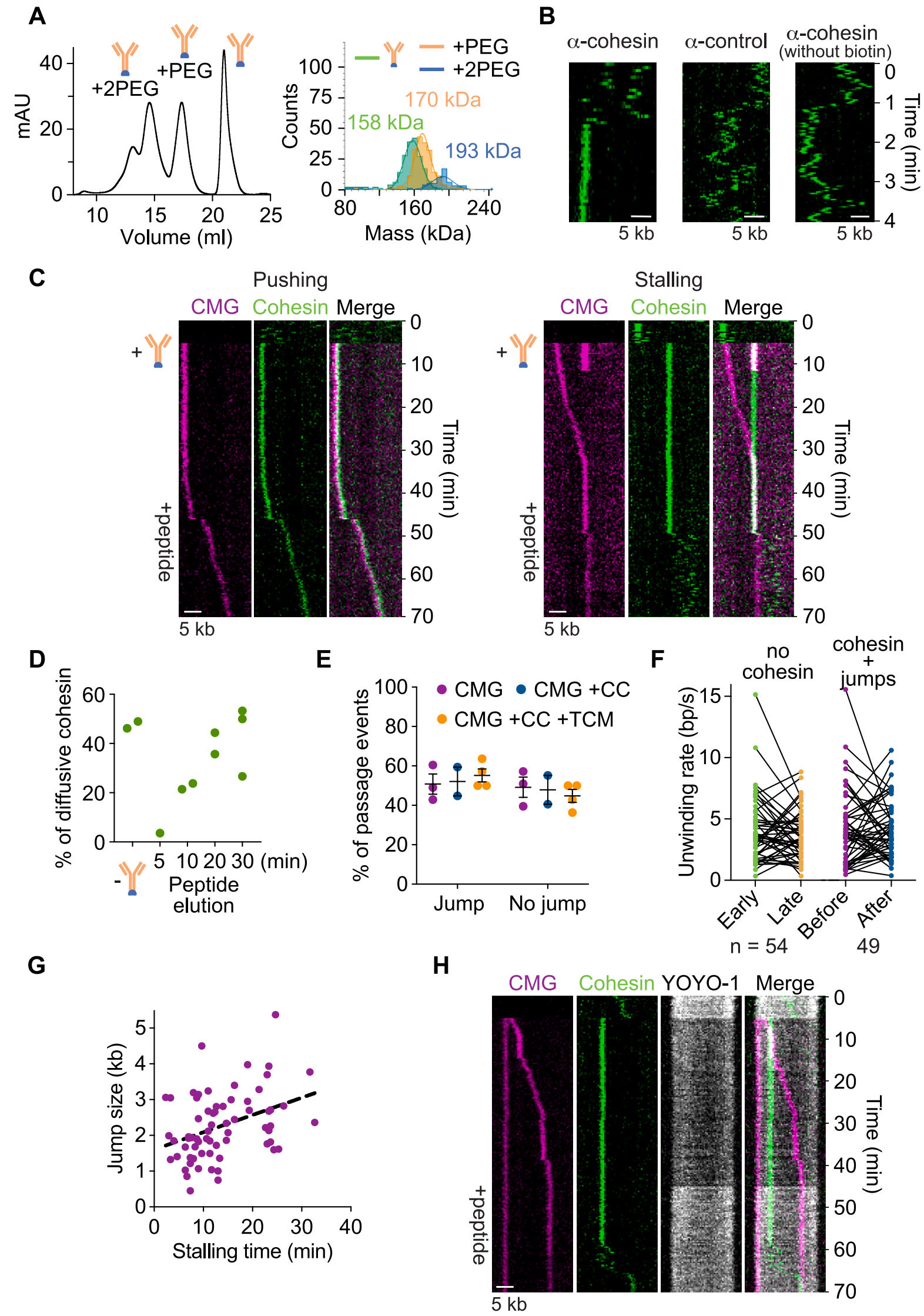

Figure S3. CMG passing immobilized cohesin, related to Figure 3

(A) Size exclusion chromatography and corresponding mass photometry profiles of functionalized V5 antibody after reacting with 20 kDa (~150 nm) biotin-PEG-succinimidyl valerate. Molecular weights correspond to unreacted, single-, and double-labeled antibody, respectively. Fractions from the single labeled peak were pooled.

(B) Representative kymographs of DNA-bound cohesin after introducing the biotin-PEG-coupled V5 antibody, a biotin-PEG-coupled control antibody, or the V5 antibody lacking the biotin-PEG tag.

(C) Representative kymographs of CMG pushing a previously immobilized cohesin (left) and of CMG stalling at immobilized cohesin (right).

(D) Percentage of diffusive cohesin observed before antibody immobilization and at the indicated times after peptide addition. Each value represents the fraction of diffusive over static cohesin in one field of view, obtained in biological replicate experiments. Always static cohesins are likely surface- rather than DNA-bound molecules.

(E) Classification of CMG passage events with or without discernible jump, in the absence or presence of the indicated cohesion establishment factors. Each data point is the result of a biological replicate. The black lines represent the mean and standard error.

(F) Segment translocation rates for CMG before and after jumps during cohesin encounters. For comparison, CMG translocation rate changes while progressing on cohesin-free DNA are shown. CMG translocation speed varies over time, but cohesin jump events did not have a directional effect on translocation speed. *n* is the number of analyzed translocation events.

(G) CMG jump size upon encounter of immobilized cohesin is plotted against the recorded stall time. Each data point represents one passage event. The black dashed line represents a simple linear regression (R^2 = 0.1432, *p*(slope deviates from zero) = 0.0016).

(H) Representative kymograph of CMG passing immobilized cohesin where the CMG stalling is short, and no YOYO-1 staining increase is apparent during the stalling time.

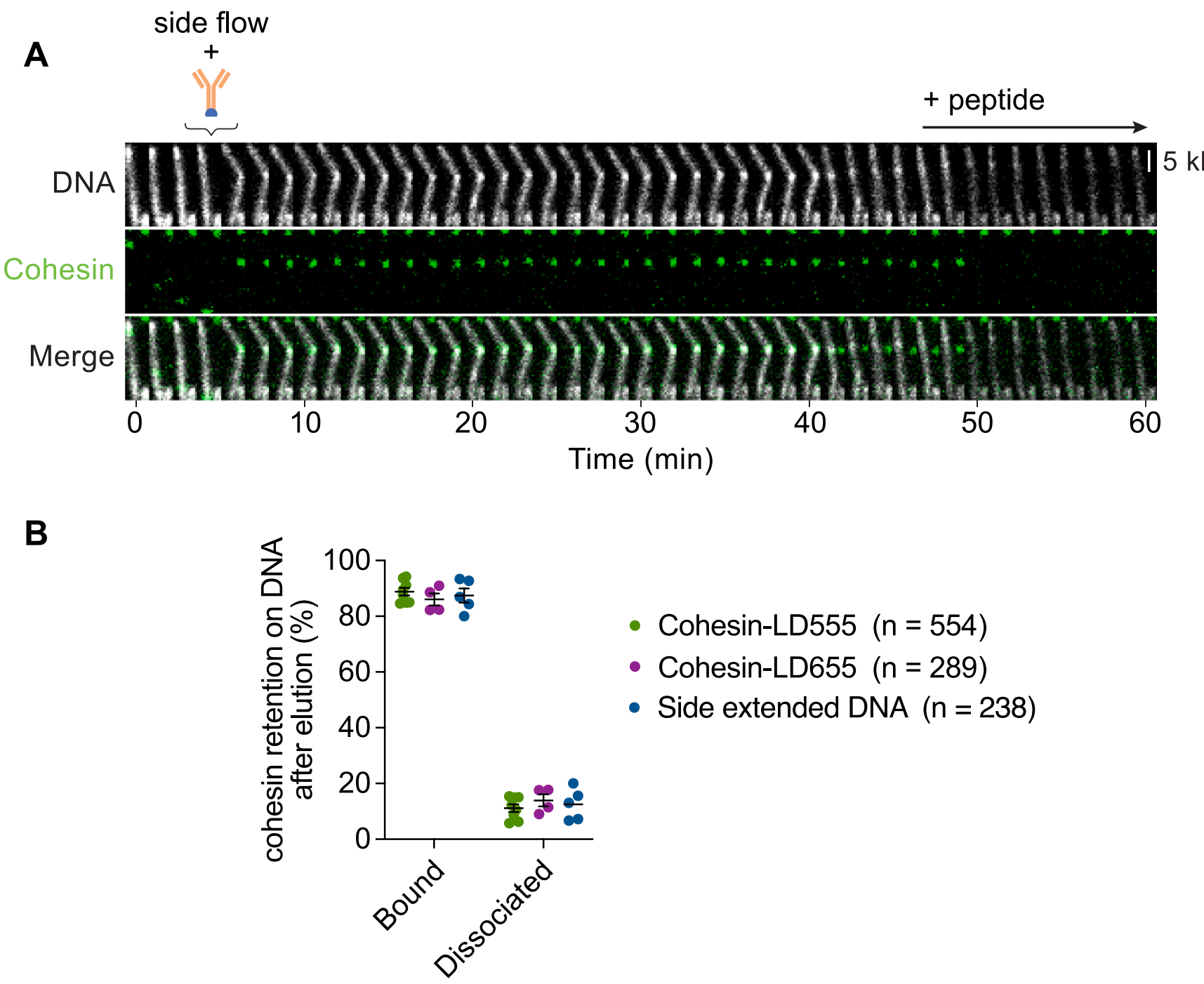

Figure S4. Cohesin anchoring and release of side-flow-stretched DNA, related to Figure 4

(A) Representative montage of side-flow-stretched DNA (side flow and antibody were applied where indicated, though that time is omitted from the montage). Cohesin maintained the V-shaped DNA geometry but released the DNA at ~40 min, presumably due to spontaneous unloading. Upon V5 peptide addition, cohesin was eluted from the surface (at ~50 min).

(B) Quantification of cohesin stability on stretched DNA. Cohesin was immobilized in the presence of side flow (side extended DNA) or without (cohesin-LD555 and cohesin-LD655). Following peptide elution, we counted the fraction of cohesins that remained DNA-bound or that dissociated. The analysis revealed that, irrespective of DNA geometry, around 10% of cohesins spontaneously unloaded from the DNA during these procedures.

CellPress
OPEN ACCESS

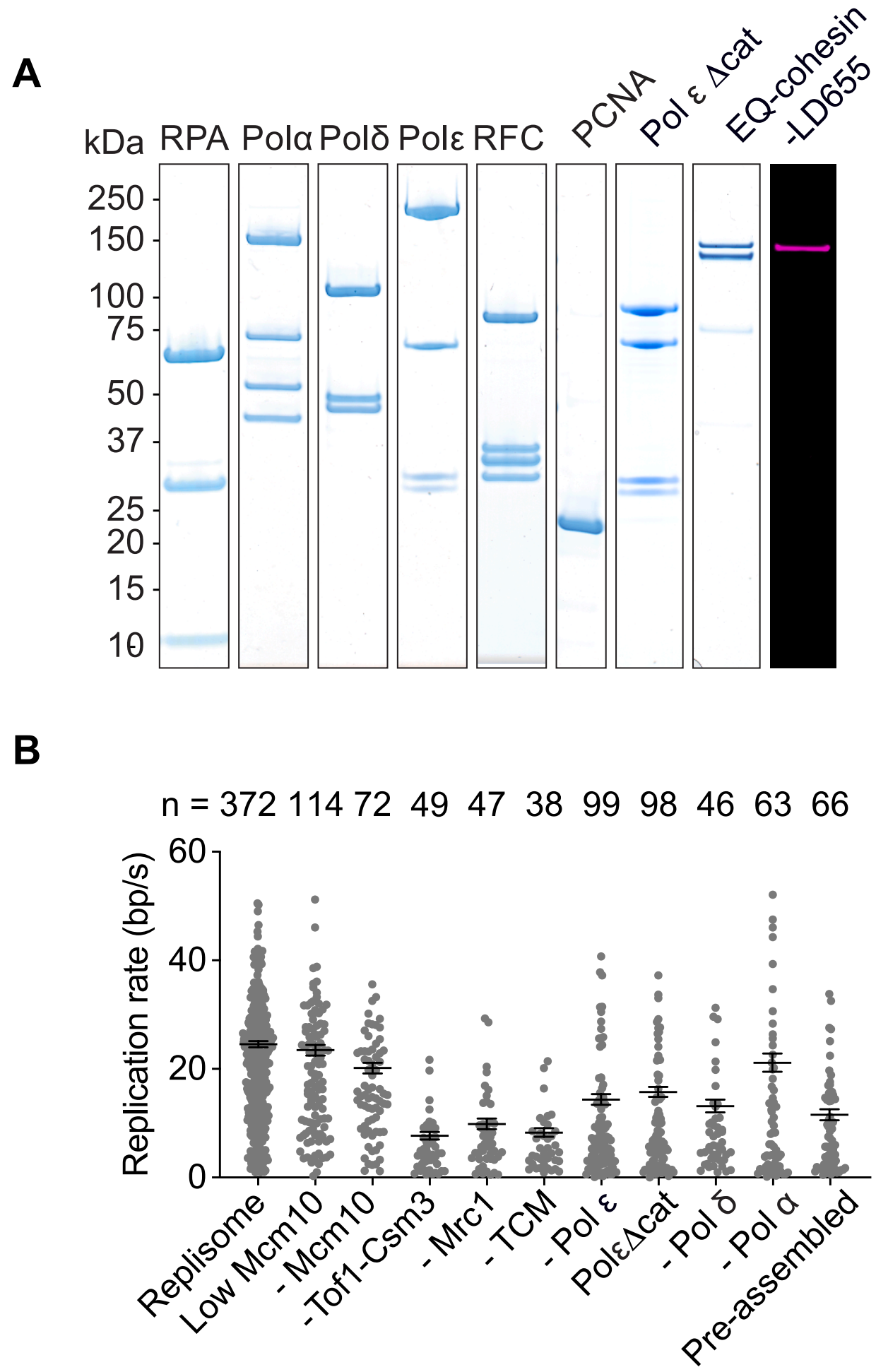

Figure S5. Single-molecule replisome-cohesin encounters, related to Figures 5, 6, and 7

(A) Purified budding yeast RPA, Polα, Polδ, Polε, RFC, PCNA, Polε Δcat, and EQ cohesin were analyzed by SDS-polyacrylamide gel electrophoresis and Coomassie blue staining (as well as by in-gel fluorescence in the case of LD655-labeled EQ cohesin).

(B) Quantification of DNA replication rates of complete replisomes and the indicated variants. Each data point represents a DNA replication segment. Black lines and error bars represent the weighted mean and standard error. *n* is the total number of analyzed segments.

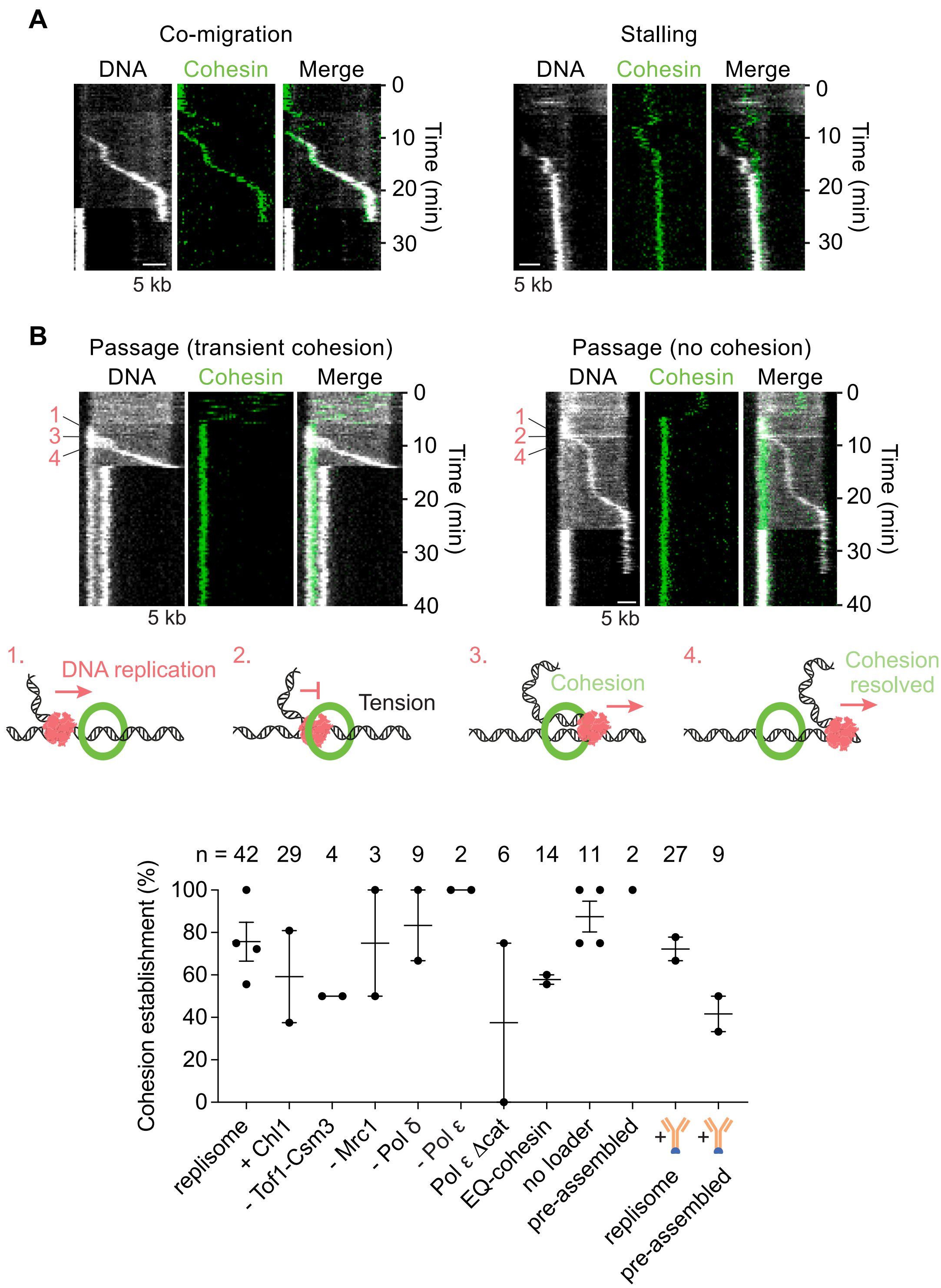

Figure S6. Replisome encounter with immobilized cohesin, related to Figures 5, 6, and 7

(A) Representative kymographs of replisome-cohesin encounters that resulted in cohesin-replisome co-migration (left) or in replisome stalling (right)

(B) Representative kymographs of replisome-cohesin encounters that resulted in cohesin passage with only transient cohesion establishment (left) or without cohesion establishment (right). The schematics illustrate how the free leading-strand DNA end might slip through cohesin rings. Quantification of successful cohesion establishment during replisome-cohesin passage events for the various indicated experimental conditions is also shown. Each data point represents a biological repeat experiment. Black lines and error bars represent the mean and standard error, and *n* is the total number of replisome-cohesin passage events per condition.

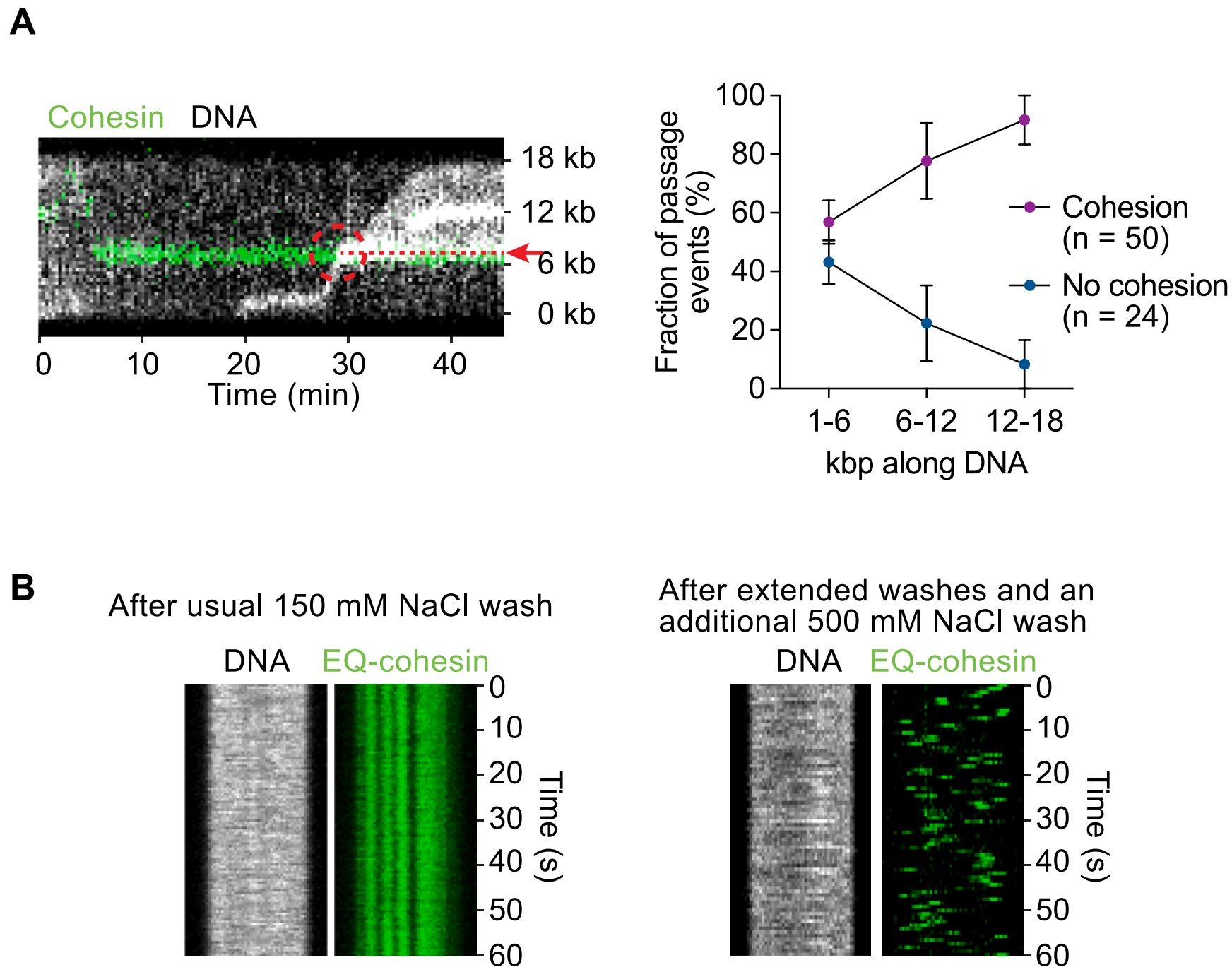

Figure S7. Cohesion establishment during replisome passage, related to Figures 5 and 7

(A) Cohesion establishment rates as a function of replisome-cohesin encounter location along the template DNA. A representative kymograph of cohesion establishment following passage of a replisome is shown. The initial encounter point is circled. The red dotted line and arrow indicate the encounter location along the template DNA, which was divided into three equal segments corresponding to 0–6, 6–12, and 12–18 kb, respectively. The frequency of successful cohesion establishment in each segment was then established and plotted. The individual results from biological repeat experiments are shown, with the mean and standard errors indicated. The total sample size (*n*) for each outcome category is given.

(B) Representative kymographs of DNA association by EQ cohesin. Following a standard loading and washing (250 mM potassium glutamate + 150 mM NaCl) protocol, EQ-cohesin complexes remained stably bound to the DNA (left), presumably having adopted an ATP-bound DNA gripping state.[15] After continuing, as well as more stringent washes (250 mM potassium glutamate + 500 mM NaCl), most cohesin eventually dissociated from the DNA. The few remaining cohesin molecules showed diffusive mobility typical of topologically loaded cohesins.

See the STAR Methods for further details.
