# Supplementary material for: Replisome passage through the cohesin ring
Source: Cell. Author manuscript; Available in PMC 2026 Aug 26. (PMC7619401; doi:10.1016/j.cell.2025.08.028)
Supplement: Supplementary file 2 [file EMS217828-supplement-Table_S1.pdf]

**Cell, Volume *188***

## Supplemental information

# Replisome passage through the cohesin ring

**Samson Glaser, Maxim I. Molodtsov, John F.X. Diffley, and Frank Uhlmann**

**Table S1. Yeast strains used in this study, related to the STAR Methods**

All budding yeast *S. cerevisiae* strains were of the W303 background.

| Strain No. | **Expressed Protein Name**<br>Genotype | Source |
| --- | --- | --- |
| Y6375 | **CMG-S6**<br>*MAT***a/α**<br>*pep4::KanMx4/pep4::KanMx4*<br>*bar1::Hph-NT1/bar1::Hph-NT1*<br>*trp1-1::TRP1pJF3 (MCM5-GAL1-10-MCM4)/*<br>*trp1-1::TRP1pJCZ1 (PSF1-GAL1-10-SLD5)*<br>*leu2-3::LEU2pJF4 (MCM7-GAL1-10-MCM6)/*<br>*leu2-3::LEU2pJCZ2 (PSF2-GAL1-10-PSF3)*<br>*ura3-1/ura3-1::URA3pRS306/MCM2-GAL1-10-CBP-TEV-MCM3)*<br>*his3-11,15/his3-11,15::HIS3pSMG1*<br>*(Cdc45iFLAG2-**S6**-GAL1-10-GAL4)* | This study |
| Y5278 | **Scc2-Scc4 cohesin loader**<br>*MAT***a**<br>*pep4::HIS3*<br>*ade2-1::ADE2pGAL-SCC2-2ProtA*<br>*trp1-1::TRP1pGAL-SCC4-HA3* | Minamino et al., 2018 |
| Y5345 | **Tetramer Cohesin-SNAP**<br>*MAT***a**<br>*pep4::HIS3*<br>*wpl1::LEU2*<br>*eco1::KANMX6*<br>*pGAL-GAL4*<br>*ade2-1::ADE2pGAL1-10-SMC1-PK*$_3$<br>*trp1-1::TRP1pGAL-OptSCC1-HIS*$_6$*-HA-3C-ProtA;*<br>*pGAL-SMC3-SNAP*<br>*ura3-1::URA3pGAL1-10-Scc3-Myc* | This study |
| Y7167 | **Tetramer EQ-cohesin-SNAP**<br>*MAT***a**<br>*pep4::HIS3*<br>*wpl1::LEU2*<br>*eco1::KANMX6*<br>*pGAL-GAL4*<br>*ade2-1::ADE2pGAL1-10-**SMC1(E1158Q)**-PK*$_3$<br>*trp1-1::TRP1pGAL-OptSCC1-HIS*$_6$*-HA-3C-ProtA;*<br>*pGAL-**SMC3(E1155Q)**-SNAP*<br>*ura3-1::URA3pGAL1-10-Scc3-Myc* | This study |
| Y5562 | **Chl1**<br>*MAT***a**<br>*pep4::HIS3*<br>*ade2-1::ADE2pGAL1-10-optimised Chl1-FLAG-2ProtA* | Shrestha et al., 2023 |
| YAE31 | **RPA**<br>*MAT***a**<br>*bar1::Hph-NT1* | Yeeles et al., 2015 |

| | | |
|---|---|---|
| | *pep4::KanMX*<br>*his3-11::HIS3pGAL1-10-CBP-Tev-RFA1+Gal4*<br>*ura3-1::URA3pGAL1-10-RFA2+RFA3* | |
| YAE34 | **Polymerase δ**<br>*MAT***a**<br>*bar1::Hph-NT1*<br>*pep4::KanMX*<br>*ura3-1::URA3pGAL1-10 POL31+POL3*<br>*his3-11::HIS3pGAL1-10 Pol32-CBP+Gal4* | Yeeles et al., 2017 |
| YAE40 | **Ctf4**<br>*MAT***a**<br>*bar1::Hph-NT1*<br>*pep4::KanMX*<br>*his3-11::HIS3pGAL1-10-CBP-Tev-Ctf4+Gal4* | Yeeles et al., 2015 |
| YAE41 | **RFC**<br>*MAT***a**<br>*bar1::Hph-NT1*<br>*pep4::KanMX*<br>*ura3-1::URA3pGAL1-10 RFC2+CBP-RFC3*<br>*Trp1-1::TRP1pGAL1-10 RFC4 + RFC5*<br>*his3-11::HIS3pGAL1-10 RFC1+Gal4* | Yeeles et al., 2017 |
| YAE48 | **Tof1-Csm3**<br>*MAT***a**<br>*bar1::Hph-NT1*<br>*pep4::KanMX*<br>*ura3-1::URA3pGAL1-10-CBP-Csm3 +Tof1* | Yeeles et al., 2017 |
| YAE71 | **Mrc1**<br>*MAT***a**<br>*bar1::Hph-NT1*<br>*pep4::KanMX*<br>*his3-11::HIS3pGAL1-10-Mrc1-TEV-3xFLAG-NAT+Gal4* | Yeeles et al., 2017 |
| YAE95 | **Polymerase α**<br>*MAT***a**<br>*bar1::Hph-NT1*<br>*pep4::KanMX*<br>*ura3-1::URA3pGAL1-10 CBP-Tev-Pri1+Pri2*<br>*trp1-1::TRP1pGAL1-10 Pol1+Pol12* | Yeeles et al., 2015 |
| YAJ2 | **Polymerase ε**<br>*MAT***a**<br>*bar1::Hph-NT1*<br>*pep4::KanMX*<br>*trp1-1::TRP1pGAL1-10 Pol2+Dpb4-CBP*<br>*ura3-1::URA3pGAL1-10 Dpb2+Dpb3* | Yeeles et al., 2015 |
| YAJ25 | **Polymerase εΔcat**<br>*MAT***a**<br>*bar1::Hph-NT1*<br>*pep4::KanMX*<br>*trp1-1::TRP1pGAL1-10* ***Pol2Δ1262****+Dpb4-CBP*<br>*ura3-1::URA3pGAL1-10 Dpb2+Dpb3* | (Yeeles et al., 2017) |

**Table S2. Oligonucleotides used in this study, related to the STAR Methods**

| Oligo No. | Sequence |
|---|---|
| 160Ld | /Phos/ACC GAT GTG GTA GGA AGT GAG AAT TGG AGA GTG TGT TTTTTT TTT TTT TTT TTT TTT TTT TTT TTT TTT TTT TTT GAG GAA AGAATG TTG GTG AGG GTTGGG AAG TGG AAG GAT GGG CTC GAG AGGTTT TTT TTT TTT TTT TTT TTT TTT TTT TTT T*T*T*T |
| 99Lg-biteg | /BITEG#/TTT TTT TTT TTT TTT TTT TTT TTT TTT TTT TTT TTT TTT TTT TTTTTT TTT TTT TTT TTT CAC ACT CTC CAA TTC TCA CTT CCT ACC ACA TCG GTC GAT |
| Fork-primer | CCT CTC GAG CCC ATC CTT CCA CTT CCC AAC CCT CAC C |
| blockLd-biteg | /BITEG#/CTG AAA TGC CAC CTA TAG CTG CGA CTC ATG |
| blockLg | /Phos/AGT CGC AGC TAT AGG TGG CAT TTC AG |
| 50duplex-lead | GAGACCGAACGATCCTGTAATGTCCTAGCAAGCCAGAATTCG GCAGCGTCTTTTTTTTTTTTTTTTTTTTTTTTTTTTTTTTTT*T*T* T*T*T*T |
| 50duplex-lag | TTTTTTTTTTTTTTTTTTTTTTTTTTTTTTTTTTTTTTTTTGACGCT GCCGAATTCTGGCTTGCTAGGACATTACAGGATCGTTCGGTCT C |
| 50duplex-lag-FAM | TTTTTTTTTTTTTTTTTTTTTTTTTTTTTTTTTTTTTTTTTGACGCT GCCGAATTCTGGCTTGCTAGGACATTACAGGATCGTTCGGTCT C[3'-Cy5] |

* 3’-phosphorothioate bonds, protecting against endonuclease activity.

# Biotin-triethyleneglycol, increasing the oligo–biotin distance to 15 atoms for reducing steric hindrance.
